# Intriguing Chloride: Involvement of Chloride Ions in Proton Transfers

**DOI:** 10.3390/molecules27041401

**Published:** 2022-02-18

**Authors:** Viktor Pilepić, Cvijeta Jakobušić Brala, Stanko Uršić

**Affiliations:** Faculty of Pharmacy and Biochemistry, University of Zagreb, A. Kovačića 1, 10000 Zagreb, Croatia; cjakobus@pharma.unizg.hr

**Keywords:** chloride, proton transfer, kinetic isotope effects, ion pair intermediates

## Abstract

The proton transfer from carbon to a chloride ion and the proton transfer to a molecule of water promoted by chloride ions in the acid-catalyzed formation of hydroxamic acids from aldehydes and substituted nitrosobenzenes in mixed solvents have been proposed based on experimental and theoretical investigations. The formation of uncommon contact ion pairs consisting of the nitrosocarbinolic cation intermediate and a chloride anion, followed by the proton transfer from a C-H moiety of the cation intermediate, has been proposed. The influence of chloride on the proton transfer to a water molecule of the solvent-separated nitrosocarbinolic-cation–chloride ion pair was investigated too. The insights are based on the obtained kinetic and other evidence with regard to (1) influences of chloride anions on the observed reaction rates and primary kinetic isotope effects (PKIE) in the reaction; (2) the observed variation of the PKIE-s and rates of the reaction when perchlorate anions are present along with the chloride ions; and (3) the consideration of a model of the nitrosocarbinolic-cation-intermediate—chloride ion pair and transition structure for the proposed proton transfers based on the ab initio calculations.

## 1. Introduction

Chloride is well known to be essential for many systems and processes in biology, and the dependence of the photosynthetic oxygen evolving system (PSII) on the chloride anion [1,2,3] is a striking example. However, it could seem strange that, to date, only two enzymes, α-amylase, and angiotensin-converting enzyme (ACE), along with PS II are known to require chloride as a cofactor. Chloride is most effective for the optimal photosynthetic oxygen evolution activity of PSII, although under some circumstances it might be replaced with bromide and some other anion. In Photosystem II, the chloride ion is proposed to be a regulator of proton transfer through the stabilization of an intermediary protonated cluster of water formed during S1 to S2 transition. The mechanism by which chloride influences the efficiency of photosynthetic oxygen evolution has yet to be completely understood. The distinctive role of chloride in the process is considered to be an important, fundamental issue in biological chemistry. However, the unusual role of chloride ions is not uncommon, even in proton-coupled electron transfer processes (PCET), which are reactions that have retained some features of proton transfers. Thus, for example, in the PCET interaction of ascorbate with hexacyanoferrate (III) in water, the chloride ions can assist in moving the reaction in the so-called deep tunnelling regime [4,5]. A further example of the unusual characteristics of chloride is the effect of chloride ions in aiding the stabilization of protonated transition states in acid-catalyzed dehydration reactions of importance for the relevant “green chemistry” [6].

The question of the involvement of chloride in proton transfer in solution chemistry can be somewhat enigmatic [6,7,8,9], although, as expected, different in some aspects from those in enzymatic environments. It is well known, on the other hand, that proton transfers are among the most important and common fundamental processes in all of chemistry and biochemistry [10,11,12,13,14,15,16,17,18,19,20,21,22]. A great number of related phenomena studied, for example the proton transfers in DNA and related systems [11,12,13], the proton coupled electron transfer processes [14,15,16,17,18], and the multiple proton transfers [19], illustrate the broadness of the field. A majority of proton transfers in chemistry and biochemistry are probably between the electronegative atoms. However, the proton transfers involving carbon [20,21], phosphorus, sulphur, and other atoms are of considerable interest too [18,22].

In contrast, questions have remained with regard to the relatively less investigated, but important matter of involvement, of chloride ions in proton transfers in solution. Earlier, reports [7,8,9] appeared dealing with the question of the possible involvement of the chloride ion in proton transfer from carbon. Thibblin et al. [7] proposed that leaving chloride in certain elimination reactions at tertiary carbon abstracts the β-proton from the C-H bond within the carbocation–chloride contact ion pair in an acetonitrile–water solvent. In the paper by Moss, Sauers, and coworkers [9] that appeared two years later, essentially the same question was addressed, though within a different context i.e., the investigation of the fragmentation of 2-norbornyloxychlorocarbenes in acetonitrile. Investigating the acid-catalyzed interactions of the aldehyde carbonyl group with C-nitroso compounds [8,23,24,25,26,27,28,29,30], we have observed [8] the unusually interconnected salt effects and the kinetic isotope effects in the reactions in mixed solvents in the presence of chloride ions. The proton transfer to a chloride anion in the process was proposed [8], where the transfer to chloride within the **1**,chloride ion pair occurs in order to rationalize the effects. However, a possibility can be envisioned where the chloride anion is only participating in a stabilization of the transition state for a proton transfer to the water molecule, somewhat similarly as in the above-mentioned acid-catalyzed dehydration reactions [6]. Moreover, in the absence of water, we have obtained [29] evidence for the proton transfer from carbon to a chloride ion in the same reaction in a 99.9% acetonitrile solution where the isotope effect effectively vanished. The situation in the mixed solvents containing a polar aprotic component and more than traces of water as well as more than one kind of anion is considerably different, however, especially concerning the solvation/hydration of the chloride ions and the formation of various ion pairs. Additionally, such mixed solvents could bear more similarities with the biological environments than acetonitrile alone; molecules of water are involved in fundamentally important proton transfers in the PS II system, for example. Finally, mixed solvents are of considerable importance to “green chemistry” [6,31].

We have now obtained the evidence of the relevance to elucidate the question about the proton transfer to chloride in the above-mentioned system [8]; furthermore, the insights obtained can explain an involvement of chloride in the proton transfer to a water molecule in the solvent-separated ion pair containing the reaction intermediate and chloride ion in the reaction system, a situation similar (to some extent) to the effects of chloride in some “green chemistry” dehydrations [6]. The evidence is comprised of: (1) the effect of a chloride anion and ion pairing phenomena on the observed rate constants and the observed primary kinetic isotope effects (PKIE) that suggest a proton transfer to chloride; (2) the effects of various anions that are simultaneously present in the reaction on the observed reaction rate constants and PKIE-s; and (3) the consideration of a model nitrosocarbinolic-cation–chloride contact ion pair based on the ab initio calculations, along with the consideration of the possible interaction of a C-H moiety on the nitrosocarbinolic cation with chloride and water within the contact- and solvent-separated ion pairs, respectively, as well as the corresponding transition structures for the proposed proton transfers.

The evidence reported herein is basically in support of the initial hypothesis, but offers a broader perspective with regard to the cases of uncommon intermediate–chloride ion pairs and the subsequent transition states where chloride can have crucial roles in the reactions in a water–organic-cosolvent system. Important in the context, the experiments show that chloride anions can be involved not only in the proton transfer from carbon to chloride; under some reaction conditions, the chloride ion can have a promoting role for proton transfer to the water molecule.

**Scheme 1:**
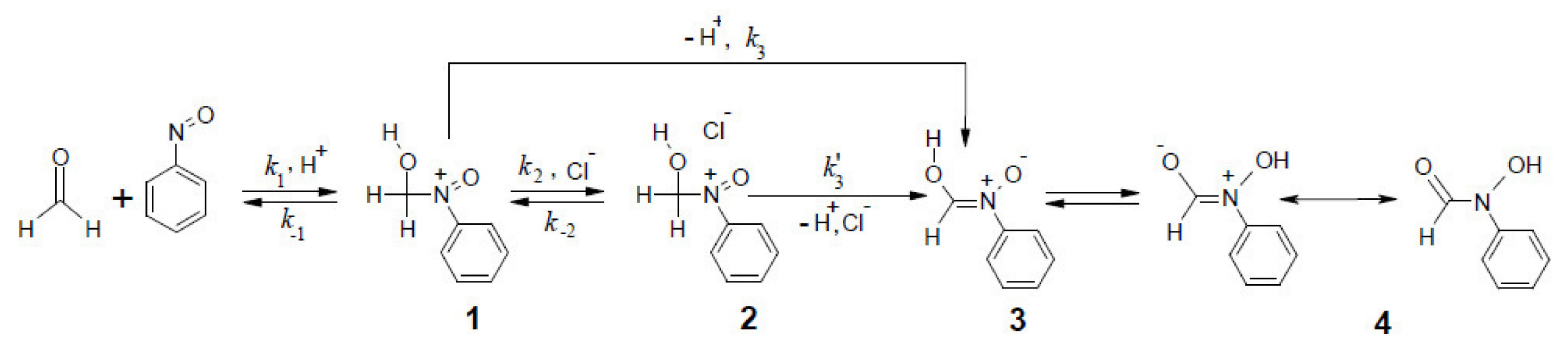
The formation of the N-phenylhydroxamic acids.

It is also worth noting that the reactions lead to the formation of the N-phenylhydroxamic acids (e.g., **4** in Scheme 1). Hydroxamic acids are compounds of considerable chemical, biochemical, pharmaceutical, and industrial significance. The acids have numerous applications, such as siderophores and enzyme inhibitors [32,33].

## 2. Results and Discussion

**Figure 1:**
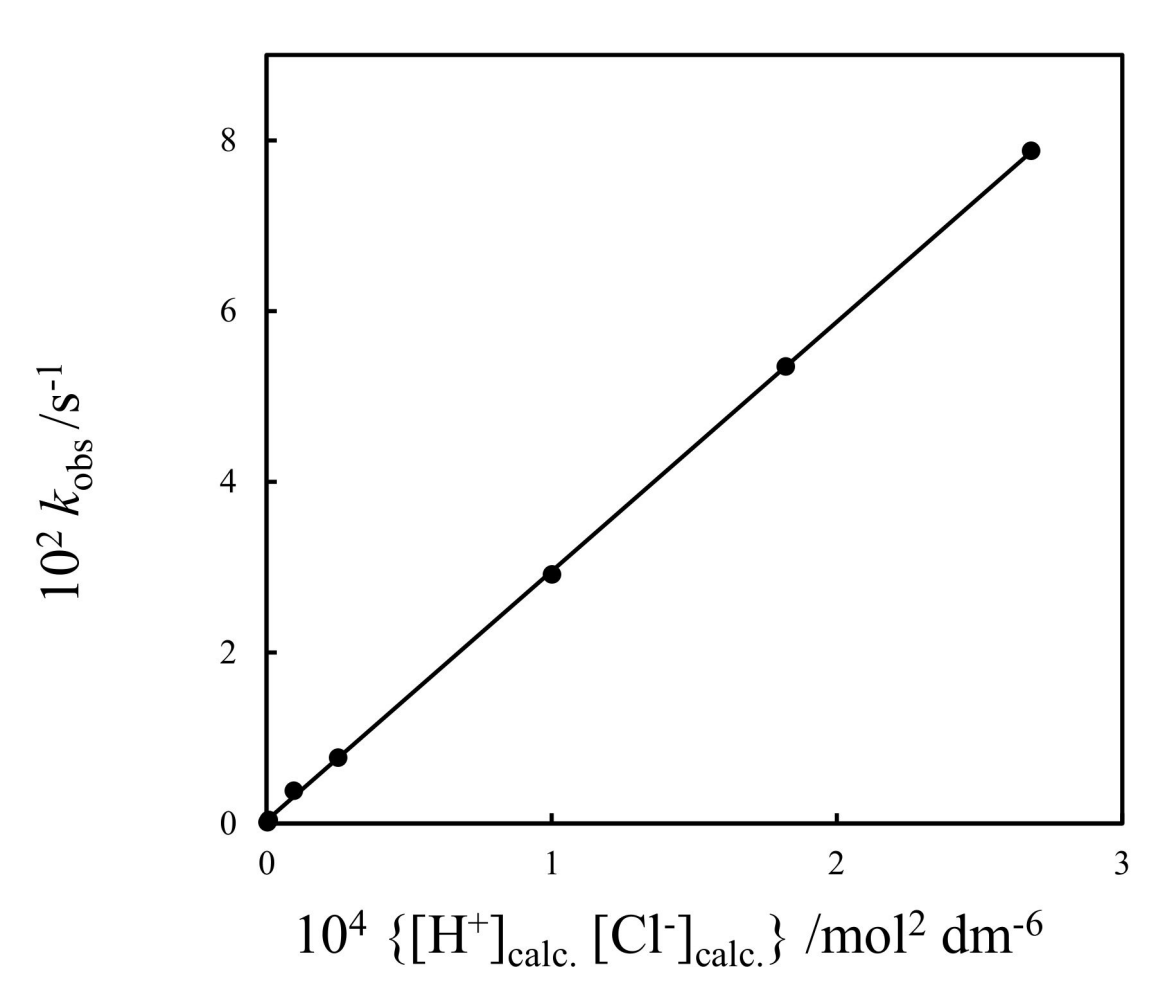
The dependence of the *k*_obs_ for the reaction of formaldehyde with nitrosobenzene in an acetonitrile-water (92.6% acetonitrile) solution on the effective concentration of the chloride anion. The points are an average of at least 3–4 runs at 25 °C, 0.200 M HCHO, and 0.0005–0.07 M HCl, *k*_obs_ = *k* [HCHO]_tot_ [H^+^]. The concentrations of the chloride anion were calculated from the concentrations of the HCl added, taking the value of 2.0 × 10^2^ M^−1^ for the association constant of HCl in a 92.6% acetonitrile-water solution at 25 °C (see Table S2).

**Figure 2:**
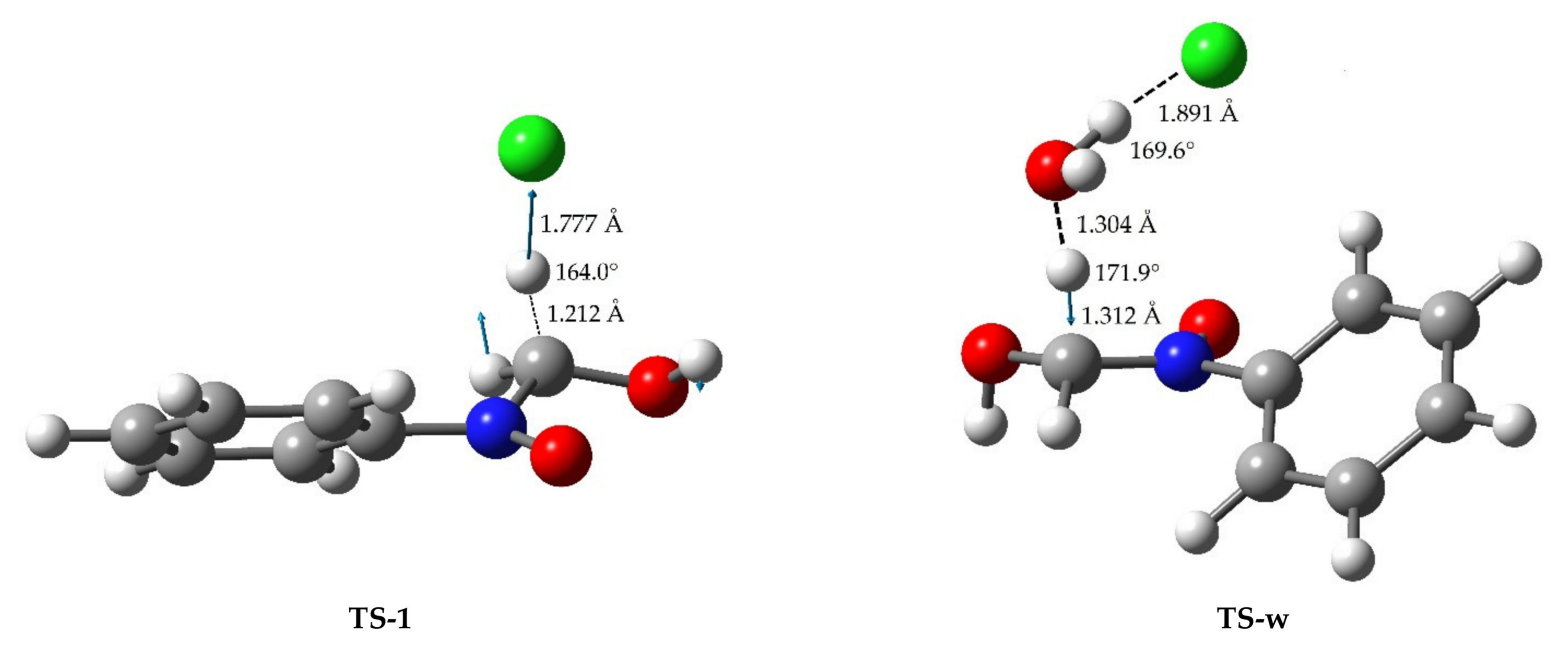
Transition structures for the proposed proton transfer from the nitrosocarbinolic cation C atom to a chloride ion in reaction step **2**→**3** within the corresponding **1**,chloride contact ion pair (**TS-1**) and to a water molecule within a corresponding **1**,chloride solvent shared ion pair (**TS-w**) obtained from ab initio calculations. The Gibbs activation energy for reactions from the corresponding ion pair are +24.6 kJ mol^−1^ and +56.0 kJ mol^−1^ for **TS-1** and **TS-w**, respectively. The normal mode displacement vectors shown (blue arrows) for the unique imaginary frequency of 946*i* cm^−1^ for transition structure **TS-1** are associated with a motion of both the H atoms of the CH_2_ moiety of the nitrosocarbinolic cation. In the case of **TS-w,** the normal mode displacement vector for the unique imaginary frequency of 1546*i* cm^−1^ is associated mainly with a motion of the H atom.

The results of the investigation are summarized in Table 1, Table 2 and Table 3, Figure 1 and Figure 2, Tables S1–S5, and Figures S1–S18. Table 1 and Table 2 comprise the observed rate constants and primary kinetic isotope effects (PKIE) in the reaction of formaldehyde and nitrosobenzene (Scheme 1) in acetonitrile–water and acetone–water mixtures caused by the small concentrations of the added Cl^−^ and Br^−^ ions, as well as the corresponding results for ClO_4_^−^, HSO_4_^−^, BF_4_^−^, and Cl_3_COO^−^ ions. A few results on acetaldehyde are also added. The extent of the association of chloride, bromide, and perchlorate ions with benzyltrimethylammonium and tetramethylammonium ions in these solvents was estimated from the conductometric measurements in order to obtain additional evidence of the possible ion pairing affinity of the nitrosocarbinolic cation (see Table S2).

### 2.1. The Mechanism of the Reaction

The mechanism of the formation of hydroxamic acid **4** in water and water-rich mixed solvents (the reaction path without the chloride added in Scheme 1) has been proposed on the basis of the kinetic evidence with regard to the order of the reaction, the acid catalysis, the dependence of the observed rate constants on the Hammett σ parameters, the observed solvent isotope effects, and the substrate kinetic isotope effects, and is discussed elsewhere [23,24,25,26,27,28,29,30]. The kinetic, spectroscopic, and product analysis evidences obtained here in the case of the addition of acetonitrile into the water reaction system, as well as in the 92.6% acetonitrile–water solvent on the addition of an inert salt, reveal only the changes in the magnitude of the observed rate constants of the reaction. The observations are as follows: (*i*) changes in the spectra of the reactants and products are the same under the various reaction conditions employed (for some examples see Figures S1 and S2). Although the same spectral changes do not necessarily imply the same mechanism, we believe that the observed spectral changes corroborate the mechanism (along with the other observations, see below), rather than suggesting a mechanistic change; (*ii*) the dependencies of the observed reaction rate constants on the concentrations of the reactants and the catalyst under the various reaction conditions (Figures S3–S5) do follow the pattern reported [8,23,24,25,26,27,28,29,30]; (*iii*) the Hammett *ρ* parameter of −2.22 for the reaction in 92.6% acetonitrile has been observed (Figure S5) in accordance with the proposed nucleophilic [8,23,24,25,26,27,28,29,30] interaction of the nitroso compound. The corresponding value observed for the reaction in water [23,24,25] is −1.74. However, the observed value is consistent with the earlier interpretation for the water system; the difference is not very large, but is what could be expected as the consequence of lowering the solvent polarity if there is a polar transition state in the reaction; and (*iv*) the PKIEs between formaldehyde and [D_2_]formaldehyde of 7.58 (0.46) and 7.53 (0.38) in the reaction in 92.6% acetonitrile–water solvent with perchloric acid and sulphuric acid as the catalysts, respectively (see Table 1), are essentially the same as the corresponding value of 7.78 (0.32) that was observed for the reaction in a water solution [23,24]. The existence of the nitrosocarbinolic cation intermediate in the process is the important feature of the mechanism, and is supported by evidence obtained earlier [8,23,24,25,26,27,28,29,30].

### 2.2. The Effect of the Cosolvent on the Rate of Reaction

Initially, the rate of reaction decreases with the addition of the organic cosolvent. The observed decrease of the rate constant is what could be expected if there are polar transition states in the reaction (as indicated, for example, by the Hammett *ρ* reaction parameter) due to the possible stabilization of the transition states by dipole-dipole interactions with water molecules. The more pronounced rate decrease observed in the less polar water–acetone solvent system (Figure S3) appears to be in line with this expectation. Further, no formation of the ion pairs consisted of the nitrosocarbinolic cation intermediate, and the anion can be expected at a high fraction of water in the mixed solvent. In this context, it is worth noting that the dependence of log *k*_obs_ on the acetonitrile content in the reaction mixture containing 0.1 M of HClO_4_ is fairly linear in the range of 0–60% acetonitrile (Figure S3). A more complete interpretation of the dependence could be difficult, but it seems that the value of *k*_obs_ extrapolated for 92.6% acetonitrile from this line (3.37 × 10^−5^ s^−1^, when [H^+^] is taken as 0.1 M and [HCHO]_tot_ as 0.200 M) could be considered as the rate constant for the reaction in this solvent under the conditions when the ion-pairing contribution to the overall rate of the process would be negligible. The comparison of the rate constants observed with the extrapolated rate constant can be indicative of the significance of ion pairing in the reaction system.

### 2.3. The Case of Chloride Ions and the Ion Pairs

The effects of chloride and other anions are summarized in Table 1 and Table 2, Figure 1, and Table S1 and Figure S4. The influence of the (effective) concentration of chloride ions on the reaction rate in the solutions containing only this anion was investigated using HCl as the catalyst (Figure 1 and Table 1, entries 5 and 6). The excellent linear dependence of *k*_obs_ vs. {[H^+^]_calc._[Cl^−^]_calc_} has been obtained for the 92.6% acetonitrile–water system (Figure 1). This, and the other results (Table 2, Figure S4), reveals the dramatic changes of the rate constants observed in the presence of very small concentrations of the chloride ion, where under the conditions employed, the reaction should go almost exclusively via the ion pair intermediates (see below). Taking into account that the observed rate law for the reaction is: *rate* = *k* [HCHO][H^+^][Ph-NO], (assuming the steady-state conditions for the formation of **1**, Ph-NO = nitrosobenzene) with *k*_obs_ = *k* [HCHO][H^+^], since the total aldehyde and H^+^ concentration were in great excess over the nitrosobenzene concentration, the observed excellent linear dependence of *k*_obs_ vs. {[H^+^]_calc._[Cl^−^]_calc_} should be consistent with the involvement of the proposed nitrosocarbinolic-cation–chloride ion pair in the reaction. In addition, the dependence of *k*_obs_/[H^+^]_calc_ vs. [Cl^−^]_calc_ is also fairly linear, as expected (not shown here). More information, however, comes from the isotopic effects observed in the system containing chloride and from experiments where other anions are present along with chloride (see below).

#### 2.3.1. The Involvement of Ion Pairs

First, the observed (Figure S3, see also Table S1) sudden increase of the reaction rate constant at high organic cosolvent content (≥90% of acetonitrile) in the presence of 0.1 M ClO_4_^−^ ion (HClO_4_ is normally considered to be fully dissociated [34] under the conditions employed) seems to indicate that in the presence of the perchlorate anion in the proposed nitrosocarbinolic-cation–perchlorate ion pair (similar to **2** in Scheme 1) is formed in the process. The rate constant at 92.6% acetonitrile is three-fold greater than the corresponding rate constant in water alone (Figure S3) and twenty-fold greater than the extrapolated rate constant, assuming no ion-pairing contribution (see in Section 2.2 above and the inset in Figure S3). The formation of an ion pair like **2** (Scheme 1) between the highly unstable cationic intermediate **1** and the chloride or perchlorate anion will, in turn, increase the concentration of the intermediate, although it is now trapped within the ion pair, thus leading to an increase of the rate of the reaction. In other words, the formation of the ion pair enhances the lifetime of the highly unstable intermediate **1.** The fact that, under the same reaction conditions, the addition of perchlorate ions leads to a significant increase of the reaction rate (Table S1) corroborates the above proposal. Note that the salt concentrations were far too low to exhibit any usual salt effect [35,36,37,38,39,40] in the case. Moreover, the very surprising effect of the addition of only millimolar concentrations of PhCH_2_(CH_3_)_3_N^+^ClO_4_^−^, where the observed substantial rate constant increase of 80% with respect to the experiment without of the added perchlorate salt (see Table S1 entry 3), is significantly greater than the corresponding figure when (CH_3_)_4_N^+^ClO_4_^−^ was added (40% increase under the same conditions employed, Table S1 entry 2) suggested a more complex mechanism of the involvement of ion pairs in the process. It should be noted that the association equilibrium quotients of PhCH_2_(CH_3_)_3_N^+^ClO_4_^−^ and (CH_3_)_4_N^+^ClO_4_^−^ are essentially the same under the conditions employed (Table S2, entries 5 and 8). Expectedly, differences in solvation and solvation/desolvation effects, in combination of dipole-dipole interactions with polar transition states [40], can be of importance to the complex mechanism of the interconversion of various ion pairs, thus leading to the observed, and seemingly unexpected, kinetic results.

Second, and in principle, if there are effects of the chloride ion in the reaction (especially the PKIE-s), as experimentally observed (Table 1 and Table 2), there must be an involvement of ion pairs in the process. Namely, the first step necessary is the formation of an encounter containing the solvated, highly unstable nitrosocarbinolic-cation intermediate and the solvated chloride ion, i.e., the formation of an encounter containing the solvent-separated ion pair. The next step could be either the interconversion of the solvent-separated ion pair (SSIP) into a contact ion pair (CIP) or the diffusion away of the partners of the solvent-separated ion pair back to the solvated ions. Of course, the interconversion of the contact ion pair back to the solvent-separated ion pair should also normally be taken into account.

**Scheme 2:**
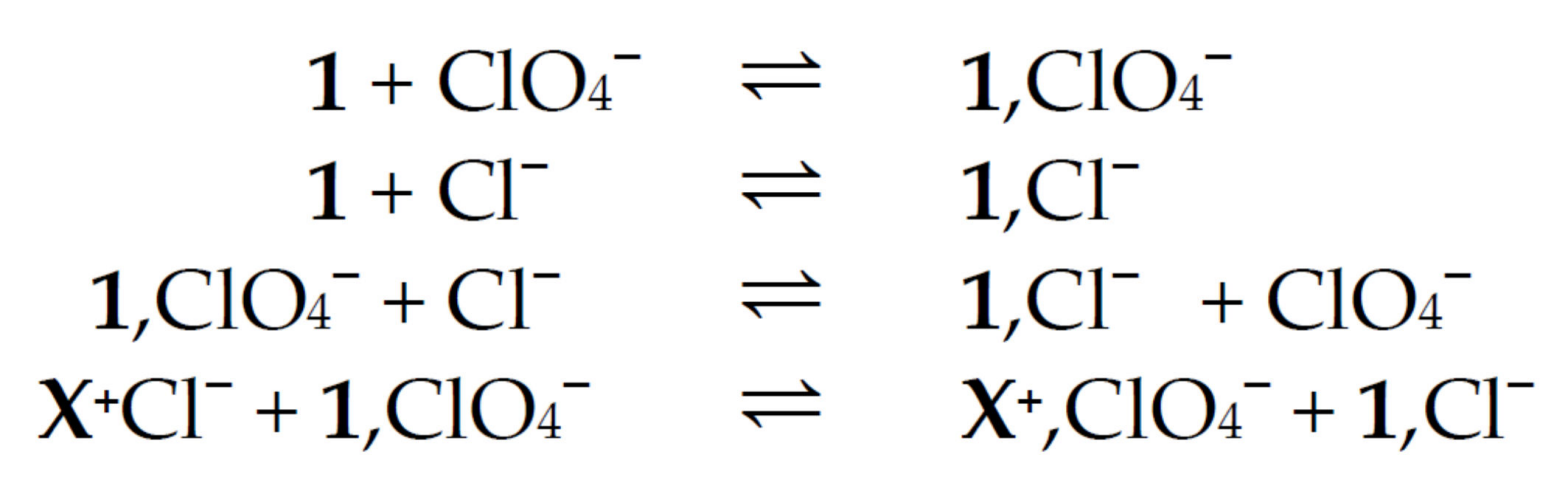
Note that **1**,Cl^−^ stands here instead of **2** in Scheme 1, for clarity. **X**^+^ = quaternary ammonium ion.

Finally, starting from the above-denoted rate law in the presence of only chloride anions in the system (see Figure 1), the rate law: *rate* = *k* [HCHO][H^+^][Cl^−^][Ph-NO] is followed, taking, however, the effective concentration of chloride ions. Although, if ion pair mechanisms are at work, different rate enhancements when both the chloride and perchlorate were present in the system should be expected. Indeed, this is what was observed, as could be easily demonstrated by the comparison of the calculated rate enhancements in these cases. Thus, for example, in the presence of 0.1 M perchlorate and 0.005 M of chloride ions (cf. entry 2, Table 2 and entry 5 in Table 1), the rate enhancement is four times greater when perchlorate ions are present as compared with the case of the presence of chloride ions only. A simplified picture of the known phenomena of ion pairing and the recombination of ion pairs [35,36,37,38,39] in this case, omitting, however, to denote different SSIP and CIP ion pairs as well as dipole-dipole interactions, could be schematized by Scheme 2 below:

Since the formation of ion pair **2** in Scheme 1, either being of SSIP or CIP type, mainly involves a coulombic interaction between the oppositely charged ions, the process is expected to be diffusion controlled (*k*_2_ in Scheme 1) and should compete with the fast breakdown of the highly unstable nitrosocarbinolic cation intermediate **1** to the reactants (*k*_−1_ in Scheme 1) successfully. The reverse process (*k*_−2_ in Scheme 1), involving a barrier for the separation of the charges, is also expected to be fast, though *k*_−2_ may be several orders of magnitude smaller than the *k*_2_ if the association affinity of the nitrosocarbinolic cation with the anions would be similar to that observed for the quaternary ions in the solvent employed (association equilibrium constants of the order of 10^2^ M^−1^, see Table S2). Hence, it does not seem to be unreasonable to expect a similar association affinity with the same anions of the nitrosocarbinolic intermediate cation, a species reminiscent of a quaternary ion. As such, the steady-state treatment involving the nitrosocarbinolic-cation–chloride ion pair intermediate should lead to the above, experimentally confirmed rate law.

#### 2.3.2. Kinetic Isotope Effects. The Involvement of Chloride in the Proton Transfer and the Role of Ion Pairs

Dramatic changes and the variation of the PKIE observed in the reaction in the presence of only small amounts of the chloride ions (cf. Table 1, entries 5 and 6) strongly suggest: (1) an involvement of the chloride ion in the proton transfer process; (2) the existence of ion pairs in the reaction; and (3) under some reaction conditions, the occurrence of proton transfer to the molecule of water within the solvent-separated intermediate **1**,chloride ion pair (cf. entries 2–4, Table 2).

First, the primary kinetic isotope effects (PKIE), *k*_H_/*k*_D_, between formaldehyde and [D_2_]formaldehyde of 7.58 and 7.53 are observed in the formation of *N*-phenylformohydroxamic acid from nitrosobenzene and formaldehyde in 92.6% acetonitrile–water reaction solution using perchloric or sulphuric acid as the catalyst, respectively. No other anions were present (cf. Table 1, entries 1 and 4). This PKIE should be consistent with the rate-controlling proton transfer from the carbon of the C-H moiety of the nitrosocarbinolic cation intermediate **1** to a molecule of water. The value of the corresponding PKIE obtained for the water solution is 7.78 [23,24]. The magnitude of this PKIE is near the maximum expected [41,42,43] for the rate-controlling proton transfer from carbon. However, the rate increase observed in the case clearly points to an involvement of ion pairs in the process. Obviously, the interaction of these anions with the C-H moiety of the nitrosocarbinolic cation in the sense that this particular anion would be operative in the proton transfer step in the reaction should be very weak or, more probably, does not occur at all, and the proton transfer should be to the water molecule within the corresponding solvent-separated ion pair.

In contrast to the case of perchlorate, a dramatic decrease of the PKIE (Table 1 entries 5, 6 and 9, and Table 2, entries 2–4) occurred in the presence of the chloride ion. In the system containing only 0.005 M of HCl (Table 1, entry 5), the PKIE of 2.02 was observed. These observations strongly suggest an involvement of chloride in the proton transfer process from the carbon of the C-H moiety of the nitrosocarbinolic-cation intermediate. More surprisingly, when 0.005 M of (PhCH_2_(CH_3_)_3_N^+^ Cl^−^) has been present in the 0.1 M perchloric acid system, the PKIE fell from 7.58 to 3.89 (Table 2, the effective chloride concentration was 0.00026 M in the case). At the same time, the observed rate constant, is more than 60-fold greater than that for the 0.1 M perchloric acid only system. This observation should indicate that less than 2% of the reaction may proceed by the mechanism operating in the perchlorate system only (cf. entry 1 in Table 1). The observation of the variation of PKIE-s in this case, in connection with the experiments where only the chloride anion has been present in the system (entries 5 and 6, Table 1), including the other related experiments quoted in Table 1 and Table 2, should require the existence of more than one kind of ion pair involved in a complex mechanism.

Second, if only proton abstraction by the chloride ion would occur (presumably within an intermediate **1**,chloride contact ion pair), the observed PKIE-s should be invariably the same in all of the experiments where the presence of chloride lead to the rate increase of one order of magnitude or greater as compared with the rate constant observed in the case of the presence of the perchlorate ion only. In other words, and inconsistent with the experiments, this would imply that the rate of separation to ions of the ion pair intermediate **2** should be negligible as compared with the rate of the proton transfer to chloride and the subsequent formation of the product, hydroxamic acid **4**.

Moreover, since it would be highly difficult to expect that the proton transfer to the chloride ion could take place within the **1**,chloride solvent-separated ion pair encounter, there must be a competition between the interconversion of the solvent-separated ion pair into the contact **1**,chloride ion pair followed by the proton transfer, and the proton transfer to the water molecule within the **1**,chloride solvent-separated ion pair on the other side. Certainly, the origin of the observed variation of PKIE-s should be traced in this way. Theoretical calculations (see below) should be in support of this picture. This relatively simple picture, although describing a much more subtle complexity, should be completed by taking into account the diffusion away of partners of the solvent-separated ion pair to free ions as well as the interconversions that include different ion pairs and even the mutual replacements of various ions/ion pairs in the second solvation shell of the intermediate **1**.

In fact, the obtained experimental results, both the variation of the PKIE-s and the insight into observed rate constants, basically require that the process of proton transfer can proceed by three different paths: (1) the proton transfer from the C-H moiety of intermediate **1** to the chloride within the **1**,chloride contact ion pair; (2) the transfer of a proton from the carbon of the C-H moiety of intermediate **1** to the water molecule within a solvent-separated **1**,chloride ion pair; and (3) the corresponding proton transfer to the water molecule within the solvent-separated **1**,perchlorate ion pair. The observed variation of the PKIE-s reveal both the involvement of chloride in the proton transfer process and the crucial importance of stabilization of the highly unstable nitrosocarbinolic-cation intermediate through the formation of the various ion pairs. The theoretical considerations can be in support of this picture (see below and in Supplementary Materials). The calculations point to the formation of a **1**,chloride contact ion pair, which leads to a more energetically favourable transition state for the proton transfer, the observed rate increase, and the dramatic changes of the PKIE-s. Hydrogen bonding C-H⋯Cl within the ion pair likely contributes to the favourable energetics of the process; the corresponding transition state should be significantly less “symmetric” as compared with the case of the proton transfer involving a water molecule. With regard to the solvent-separated **1**,chloride ion pair, as well as the **1**,perchlorate solvent-separated ion-pair, it could be expected that the effect of the highly localized negative charge on Cl^−^ may stabilize the transition state for proton transfer to the water molecule within the ion pair. This effect can be reminiscent of the proposed stabilizing effect of chloride ions on the oxocarbenium ion and the transition state for deprotonation that forms in some acid-catalyzed dehydration reactions [6].

Very interestingly, it seems that the influence of the highly localized negative charge on Cl^−^ can be operative even in the reaction in water, i.e., concentrated water solutions of chloride salts. Namely, the PKIE in the observed reaction in the case of a 3.75 M LiClO_4_ water solution is 8.6, while in the cases of 4 M LiCl and 4 M NaCl water solutions, the PKIE-s are 7.1 and 7.0, respectively [8]. The observed rate constants for chloride salts are two orders of magnitude greater than the corresponding rate constants for the perchlorates at the same time [8,30], which is reminiscent of the effects in mixed solvent systems.

A question could be whether the nitrosocarbinoliccation-intermediate–chloride ion pair **2** actually exists as the contact ion pair or if the corresponding solvent-separated ion pairs virtually completely predominate in a fast interconversion process with the former ones. Examples of equilibria between different ion pair species in electrolyte solutions are not uncommon [44]. However, since both the nitrosocarbinolic cation **1**, as well as the various possible nitrosocarbinolic ion pairs of the type **2**, are considered to be the “intermediates” existing under the steady-state conditions in the reaction, the question should be of little relevance to the proposal of the involvement of the contact ion pair intermediate **2** in the proton transfer process. The basic idea here is that a fast interconversion of various ion pairs could be possible in the mixed solvent used. Regarding this aspect, the situation would be in some sense similar to that described in the commonly accepted Winstein’s solvolysis scheme [37,38,39]. It could be added that we have obtained [29] the evidence for the proton transfer from carbon to the chloride ion in the same reaction, but under considerably different reaction conditions (organic solvent only, no water present).

#### 2.3.3. Other Kinetic Evidence

We have obtained some additional and corroborative evidence related to the above-considered questions. It can be easily demonstrated that the magnitudes of the rate constants observed when two different anions are present in the reaction system (Table 2) are always significantly greater than the sum of the corresponding expected rate constants, assuming only one reaction path picture (only one anion present in the system). The observed variation of the PKIE-s strongly corroborated this observation. The experiments involving a bromide ion (Table 1, entry 7 and Table 2, entries 5–7) should be viewed in a similar context. A reason to include the experiments with bromide include, among others, the fact that the PS II system can function with a bromide ion in place of a chloride ion.

Expectedly, a trichloroacetate anion (Table 1, entry 8) and betaine, (Table 2, entry 10) being much more basic than chloride, exhibit a change of the PKIE that indicates the involvement of the anion in the proton transfer process. The change of the PKIE for trichloroacetate is similar to the case of halide ions. It should be noted that in the case of betaine, 0.001 M of chloride ions was also present in the reaction solution. The observed decrease of PKIE is significantly greater than for other 0.001 M chloride or bromide systems (Table 2, entry 10), indicating an involvement of the betaine anion in the process. The observed increase of the rate constant appears to be expected since both chloride and betaine anions are operative in the process.

All of the remaining experiments with chloride where different cations are involved (Table 2, entries 8–11 and 13–16) show effects that are in line with the expectations related to the complexity described and explained above (see however Section 2.3.1 and Section 2.3.2). In addition, the same mechanisms could be operative in the system containing water–acetone solvent (see Table 1, entry 9 and Table 2, entry 15), although the rates of reaction are substantially reduced. This is as expected, taking into regard the interplay of less ion pairing due to an increased fraction of water, stronger water solvation, and, on the other hand, the lower polarity of the organic cosolvent. Finally, the experiments involving acetaldehyde (Table 1, entries 10 and 11 and Table 2, entry 17) also show the involvement of chloride in the reaction; this is as expected, owing to the close similarity between the two aldehyde reactions [23,25,26].

Finally, there are some similarities between the cases reported by Thibblin et al. [7] and Moss et al. [9] and our observations and conclusions. However, our observations are, in several aspects, unprecedented. In those systems [7,9], the chloride ion in the contact ion pair comes from the dissociation process, i.e., bond breaking at the substrate preceding the solvolytic substitution and elimination processes. It could be added that Bunton et al. [45] observed the promoting influence of the added chloride on the elimination involving the relatively stable carbocations many years ago. In our system, the proposed nitrosocarbinolic-cation–chloride ion pair involves the intermediate **1**, which is reminiscent of a quaternary cation and the halide ion from the dissolved inert salt or acid. The kinetic evidence [8,23,24,25,26,27,28,29,30] suggests that the nitrosocarbinolic cation is very unstable, though perhaps is of similar stability as the relatively stable carbocations, taking into regard that the cation is reminiscent of a quaternary ammonium ion.

Thibblin et al. [7,46] presented the evidence of a decreased activity of water as a proton acceptor in a 40% acetonitrile–water system and emphasized the importance of the incomplete solvation of the chloride ion within the incipient contact ion pair for the efficiency of the proton abstraction process in this case. We have also examined the case of mixed solvents containing more than a few percent of water. Significantly decreased rate constants were observed, which is in line with the expectation that an increase of the solvent polarity and partial hydration caused by the added water should decrease the extent of ion pairing in these solvents (see for example entries 2, 3 and 9, Table 1). The effect of decreased ion pairing due to the increased solvent polarity could be compensated, in part, by the increase of the fraction of dissociated chloride. However, the observed changes of the PKIE followed a similar pattern as in the mixed solvents containing less water. Hence, it seems that the above-considered ion-pairing phenomena can also be operative in the case of these mixed solvents.

### 2.4. Theoretical Calculations

The ab initio calculations, shown in detail in the Supplementary Materials, can be in support of the proposed role of chloride and ion pairs in the process. The calculations point to the formation of the **1**,chloride contact ion pair, which leads to a more energetically favorable transition state for the proton transfer in accordance with the observed rate increase and the dramatic changes of the PKIE-s. The C-H⋯Cl hydrogen bonding within the contact ion pair contributes to the favorable energetics of the process, and the corresponding transition state for the proton transfer is significantly less “symmetric” as compared with the case of the proton transfer involving a water molecule and/or perchlorate.

The molecular electrostatic potential (MEP) plotted on the electron density isosurface of the optimized geometry of the nitrosocarbinolic-cation intermediate **1** is shown in Figure S8. The most positive region of the MEP, which should be the preferred position to approach the anion, is close to the N atom and CH_2_ group. The interaction between the anion and the nitrosocarbinolic-cation intermediate should therefore occur in a vicinity of the C-H moiety, and the position of the anion would then be suitable to form a hydrogen bond during the process. The optimized structures and selected distances and angles obtained from ab initio calculations for the **1**,chloride contact ion pair **2** (2-CIP-1 and 2-CIP-2), **1**,chloride solvent-shared ion pair (2-SSIP), **1**,perchlorate contact ion pair (CIP-p1 and CIP-p2), and **1**,perchlorate solvent-shared ion pair (SSIP-p) are shown in Figures S9, S12, S14 and S16, respectively. The obtained Gibbs energy of −69.3 kJ mol^−1^ for the formation of the **1**,chloride contact ion pair (2-CIP-1) is more favorable than the Gibbs energy of −43.9 kJ mol^−1^ for the formation of the **1**,chloride solvent-shared ion pair (2-SSIP) and much more favorable than the Gibbs energies of −17.2 kJ mol^−1^ and +1.4 kJ mol^−1^ obtained for the formation of the **1**,perchlorate contact (CIP-p1) and **1**,perchlorate solvent-shared (SSIP-p) ion pairs, respectively. The noncovalent interaction (NCI) analysis of the **1**,chloride contact ion pairs, Figure S10, indicate that weak dispersive and hydrogen-bond-like interactions involving the chloride ion were present. In addition, the distances and angles between the hydrogen of the C-H and O-H and the chloride ion in the structures, Figure S8, are favorable to form a weak C-H···Cl and O-H···Cl hydrogen bond similar to that found in similar ionic liquid 1-buthyl-3-methylimidazolium cation–chloride ion pair [47]. A series of the obtained structures for the **1**,chloride contact ion pair suggests that the chloride ion is highly mobile within the vicinity of the C-H bond [47].

The properties of the transition structure for the proposed proton transfer to anions or a water molecule within the corresponding ion pairs in step **2**→**3** are shown in Table 3. The transition state structure obtained for proton transfer from the nitrosocarbinolic cation C-H moiety to the chloride ion within the contact ion pair (**TS-1**) is markedly different than the structures obtained for proton transfer to water molecules (**w-TS**), to water near the chloride ion within the corresponding SSIP (**TS-w**), to the perchlorate ion within the corresponding CIP (**TS-p**), or to a water molecule near the perchlorate ion within the corresponding SSIP (**TS-wp**). The C···H distance of 1.212 Å in **TS-1** is shorter than the distances obtained for **TS-wp**, **TS-w**, **TS-p**, and **w-TS**, suggesting an early “asymmetric” transition state that could lead to the experimentally observed reduced PKIE. Further, the normal mode displacement vectors for a unique imaginary frequency of 946*i* cm^−1^ for **TS-1** is associated with a motion of the H atom between the C atom of **1** and the chloride ion as well as the adjacent H atom (Figure 2). In the cases of **TS-wp**, **TS-w**, **TS-p**, and **w-TS,** the normal mode displacement vectors for an imaginary frequency of 1400*i* cm^−1^ or more are primarily associated with a motion of only the H atom between the C atom of **1** and the acceptor, as illustrated in Table 3, Figures S12, S15, S16 and S17. The H···Cl bond distance of 1.777 Å and 1.745 Å for the transition structures of **TS-1** and **TS-2**, respectively, are shorter than the H···Cl bond distance of 1.94 Å obtained by Moss et al. [9] for the transition structure for proton transfer within a norbornyl-cation–chloride ion pair, suggesting a strong involvement of the chloride ion in this case.

The Gibbs activation energy of +24.6 kJ mol^−1^ obtained for the proton transfer from the C−H moiety of the **1**,chloride CIP is much lower than the corresponding activation energy of +74.8 kJ mol^−1^ for proton transfer to the water molecule or the values in the range from +56.0 kJ mol^−1^ to + 63.2 kJ mol^−1^ obtained for proton transfer to the perchlorate CIP or water molecule within the chloride or perchlorate SSIP, which is also in accordance with the experimentally observed increase of the reaction rate constants presented in Table 1 and Table 2. The presented results are consistent with the proposed specific role of the chloride ion in the proton transfer process within the ion pairs.

## 3. Materials and Methods

The acetonitrile and acetone used were of HPLC-grade purity (Merck). The quaternary salts used were of high-grade purity (Fluka) and were used without further purification. Formaldehyde and [D_2_]formaldehyde were from Fluka and Aldrich. Other chemicals used were of analytical-grade purity. Conductometric measurements were performed using a WTW-LF 300 conductivity meter. A HP 8453 UV-VIS spectrophotometer was used for the spectrophotometric measurements.

The apparent association constants (referred to as the concentration equilibrium quotients for the formation of a simple ion pair) of HCl and other acids employed and the quaternary salts in 92.6% acetonitrile–water solvent, *K*_assoc_ = [M^+^A^−^]/[M^+^][A^−^] related to the equilibrium M^+^ + A^−^ Ý M^+^A^−^ were estimated using the conductometric data obtained for the electrolytes in the medium (Table S2). For accurate association constants, a more complex treatment is required, but an estimate would be acceptable for the present purpose. The procedure used here starts from the arbitrary assumptions that only simple ion pairs are formed in the medium under the conditions employed, and that the correction for electrophoretic and relaxation effects could be omitted in the concentration range of 10^−5^ M to 10^−3^ M. The following experimental observations are behind the assumptions: (a) the dependence of molar conductivity Λ_m_ vs. [MA]_tot_ (the analytic concentration of electrolyte) is strongly linear (ordinarily, *r*^2^ = 0.9999) in the range of 1 × 10^−5^ M to 1 × 10^−4^ M in the medium; and (b) the deviation from the linearity is relatively small up to 1 × 10^−3^–2 × 10^−3^ M for the electrolytes. The measurements were performed using a well thermostatted glass compartment, at 25 °C. Usually, 25–30 points were taken in the 1 × 10^−5^ M to 5 × 10^−3^ M concentration range. The obtained data were then fit to the equation *K*_assoc_ = (1 − Λ_c_/Λ_o_){(Λ_c_/Λ_o_)^2^[MA]_tot_}^−1^, where Λ_c_ and Λ_o_ are the molar conductivity at the analytical concentration of the electrolyte [MA]_tot_ and the limiting molar conductivity, respectively. Good fits (ordinarily, *r*^2^ = 0.9999) of the apparent values of *K*_assoc_ (M^−1^) and Λ_o_ (S cm^2^ mol^−1^) in 92.6% acetonitrile–water have been obtained (Table S2).

Kinetics and product analysis were performed using the methods described elsewhere [8,23,24,25,26,27,28,29,30]. Pseudo-first order rate constants were obtained spectrophotometrically by following the disappearance of the absorbance of nitrosobenzene at 306 or 310 nm. Usually, concentrations of the total formaldehyde and that of the corresponding acid (HClO_4_ or HCl) were 2 × 10^2^–10^4^ times greater than the concentration of the nitroso compound. Kinetics were ordinarily initiated by adding of the appropriate amount of the solution of nitrosobenzene in acetonitrile or acetone into a previously thermostatted reaction mixture (usually 2 mL) in the stoppered cuvette and then placed in the well thermostatted cell compartment. Ordinarily, 30–100 time/absorbance points were taken in the least-square fitting procedure for processing the data and pseudo-first order kinetics were obtained in all the experiments for at least 3–4 half-lives. Kinetic isotope effects were calculated using at least 3–4 paired measurements.

All the ab initio molecular orbital calculations were performed using the GAUSSIAN 09 package [48] at MP2/6-311++G(d,p) level in a solution phase. Non-specific solvent effects have been estimated by using the polarizable continuum model (PCM) of self-consistent reaction field (SCRF) method [49] with acetonitrile as a solvent. The geometry optimizations have been started from numerous conformers of nitrosocarbinolic cation intermediates and different initial structures with anions placed near the positive region of the nitrosocarbinolic cation in the nitrosocarbinolic-cation–chloride ion pair and transition structures, respectively. The obtained results reveal a very flat potential energy surface, and tight convergence criteria were applied. The obtained stationary points were confirmed either as minima or saddle points by vibrational analysis at the same theory level. The noncovalent interaction analysis (NCI) was performed with the MULTIWFN 3.8 software [50] and the results were plotted with VMD 1.9.4 [51].

## 4. Conclusions

The kinetic isotope effects study showed that chloride ions can be involved in proton transfers in solution chemistry; the obtained evidence related to the system studied suggest the involvement of chloride anions in the proton transfers from the carbon of the nitrosocarbinolic-cation intermediate in the reaction. The proton transfers occur from the carbon to the chloride ion within the nitrosocarbinolic-cation–chloride contact ion pair and from the carbon to the water molecule within the corresponding solvent-separated ion pair.

The formation of the uncommon contact ion pair intermediates consisting of the nitrosocarbinolic-cation intermediate, a kind of ion intermediate reminiscent of the quaternary ions and the chloride anion, appears to be crucial for the involvement of the chloride ions in the transition state for proton transfer from carbon to chloride in the reaction.

The formation of the ion pair intermediates led to dramatic changes of the reaction rate constants, as well as dramatic changes of the observed PKIE-s, in the presence of chloride anions in the reactions using mixed solvents with a relatively low fraction of water.

A particularly important point related to the role of chloride is the observation of some cooperative involvement of the ion in the proton transfer to the water molecule within the solvent-separated nitrosocarbinolic-chloride ion pair, a reaction path occurring along with the above proton transfer to chloride within the corresponding contact ion pair. The process takes place under certain reaction conditions and is evidenced by the variation of PKIE-s and rate analysis. The favorable thermodynamics due to desolvation, along with favorable electrostatic effects, are the most influencing factors to the phenomena observed in the systems. Another item of significance would be the fact that the proton transfer from carbon to chloride can occur even in the mixed solvent containing several moles of water.

The theoretical consideration, in support of the picture proposed, has shown that the nitrosocarbinolic-cation–chloride contact ion pair are more stable than the nitrosocarbinolic-cation–chloride solvent-shared ion pair or the nitrosocarbinolic-cation–perchlorate contact or solvent-shared ion pairs. In addition, the Gibbs activation energy for the proposed proton transfer reaction step to the chloride ion within the contact ion pair is much lower than the Gibbs activation energy for the corresponding proton transfer to the water molecule within a corresponding chloride or perchlorate solvent-separated ion pair.

These results could be of use in understanding the roles of chloride in proton transfers within related systems, for instance in mixed solvents for “green chemistry”, where electrostatic and desolvation effects obviously are of importance, and, similarly, in enzymatic systems. Therefore, when these effects can be operative, some aspects related to chloride, and described in this case, could not be excluded. Even, for example, in the PS II system; the obvious subtlety of the interplay of electrostatic and desolvation effects in the case reported here could be of use for a better understanding of the regulation of proton transfer by chloride in the PS II system, a question which is a fundamental issue in biological chemistry.

## Figures and Tables

**Table 1:** Influence of the particular anion on the observed rate constants and kinetic isotope effects in the reaction of aldehydes and nitrosobenzene ^a,b^.

	Anion	10^4^ *k*_H_/s^−1^	10^4^ *k*_D_/s^−1^	PKIE ^c^
1	ClO_4_^−^ (HClO_4_, 0.1 M) ^d^	12.36 (0.75)	1.34 (0.06)	7.58 (0.32)
2	ClO_4_^−^ (HClO_4_, 0.1 M) ^e^	1.35 (0.04)		
3	ClO_4_^−^ (HClO_4_, 0.1 M) ^d,f^	2.32 (0.06)	0.30 (0.01)	6.34 (0.17)
4	HSO_4_^−^ (H_2_SO_4_, 0.005 M)	1.22 (0.01)	0.16 (0.01)	7.53 (0.38)
5	Cl^−^ (HCl, 0.005 M)	39.30 (1.07)	19.4 (0.75)	2.02 (0.10)
6	Cl^−^ (HCl, 0.050 M)	251.6 (7.16)	135.0 (5.65)	1.86 (0.09)
7	Br^−^ (HBr, 0.005 M)	50.7 (0.50)	22.1 (0.40)	2.29 (0.05)
8	CCl_3_COO^−^ (CCl_3_COOH, 0.050 M)	1.83 (0.08)	0.44 (0.01)	4.11 (0.20)
9	Cl^−^ (HCl, 0.050 M) ^g^	5.54 (0.21)	2.37 (0.20)	2.34 (0.22)
10	ClO_4_^−^ (HClO_4_, 0.1 M) ^h^	0.40 (0.04)		
11	Cl^−^ (HCl, 0.050 M) ^h^	3.16 (0.10)		

^a^ Only one kind of the anion is present in the system. ^b^ Rate constants were expressed as the pseudo-first order rate parameters according to the observed rate law: *rate* = *k* [HCHO]_tot_ [H^+^] [Ph-NO], (Ph-NO = nitrosobenzene) and are an average of at least 3–4 runs. At 25 °C, in 92.6% acetonitrile-water, 0.100 M HClO_4_, and 0.200 M HCHO, except otherwise noted. Standard errors are in parentheses. ^c^ Primary kinetic isotope effect between formaldehyde and [D_2_]formaldehyde. ^d^ [HCHO]_tot_ = 0.259 M, [DCDO]_tot_ = 0.213 M. ^e^ In 70% acetonitrile-water. ^f^ In 92.6% acetone-water. ^g^ In 80% acetone-water, assuming no significant H^+^Cl^−^ association. ^h^ Rate constant for the reaction with 1.0 M acetaldehyde. Relative to the rate constant, with 0.100 M HClO_4_. The composition of the organic phase was 87% acetonitrile - 5.6% acetaldehyde. It was assumed that the dissociation of HCl in this medium is similar to that observed in the 92.6% of acetonitrile–water one.

**Table 2:** Influence of the anions on the observed rate constants and kinetic isotope effects in the reaction of aldehydes and nitrosobenzene ^a,b^.

	Anion	10^3^ *k*_H_/s^−1^	10^3^ *k*_D_/s^−1^	PKIE ^c^
1	Cl^−^, ClO_4_^−^ (PhCH_2_(CH_3_)_3_N^+^ Cl^−^ 0.01 M) ^d^	1.01 (0.04)		
2	Cl^−^, ClO_4_^−^ (PhCH_2_(CH_3_)_3_N^+^ Cl^−^ 0.005 M) ^e,f^	62.3 (0.08)	13.07 (0.09)	3.89 (0.06)
3	Cl^−^, ClO_4_^−^ (PhCH_2_(CH_3_)_3_N^+^ Cl^−^ 0.001 M) ^e,f^	13.43 (0.04)	2.92 (0.06)	3.76 (0.12)
4	Cl^−^, ClO_4_^−^ (PhCH_2_(CH_3_)_3_N^+^ Cl^−^ 0.0001 M)	4.40 (0.13)	0.98 (0.14)	4.49 (0.20)
5	Br^−^, ClO_4_^−^ (PhCH_2_(CH_3_)_3_N^+^ Br^−^ 0.005 M)	274.0 (5.0)	119.0 (1.0)	2.30 (0.05)
6	Br^−^, ClO_4_^−^ (PhCH_2_(CH_3_)_3_N^+^ Br^−^ 0.001 M)	32.5 (1.2)	8.44 (0.05)	3.85 (0.14)
7	Br^−^, ClO_4_^−^ (PhCH_2_(CH_3_)_3_N^+^ Br^−^ 0.0001 M)	6.65 (0.03)	1.54 (0.01)	4.32 (0.03)
8	Cl^−^, ClO_4_^−^ ((CH_3_)_4_N^+^ Cl^−^ 0.001 M)	5.35 (0.02)	1.15 (0.02)	4.65 (0.08)
9	Cl^−^, ClO_4_^−^ ((CH_3_CH_2_)_4_N^+^ Cl^−^ 0.001 M)	9.24 (0.18)	3.01 (0.09)	3.07 (0.11)
10	Cl^−^, ClO_4_^−^ (Betaine hydrochloride 0.001 M)	25.0 (0.2)	11.1 (0.2)	2.25 (0.04)
11	Cl^−^, ClO_4_^−^ (1-Buthyl-3-Methyl Imidazolium Cl^−^ 0.001 M)	7.92 (0.06)	1.87 (0.08)	4.24 (0.18)
12	BF_4_^−^, ClO_4_^−^ (1-Buthyl-3-Methyl Imidazolium BF_4_^−^ 0.001 M)	2.47 (0.03)	0.444 (0.008)	5.61 (0.12)
13	Cl^−^, ClO_4_^−^ (LiCl 0.0044 M) ^g^	9.91 (0.37)		
14	Cl^−^, ClO_4_^−^ (LiCl 0.0088 M) ^g^	14.54 (0.55)	5.44 (0.63)	2.19 (0.08)
15	Cl^−^, ClO_4_^−^ (MgCl_2_ 0.01 M) ^h^	0.171 (0.004)	0.057 (0.002)	3.01 (0.14)
16	Cl^−^, ClO_4_^−^ (MgCl_2_ 0.01 M) ^i^	0.586 (0.017)	0.182 (0.007)	3.22 (0.16)
17	Cl^−^, ClO_4_^−^ (PhCH_2_(CH_3_)_3_N^+^ Cl^−^ 0.005 M) ^j^	0.18 (0.003)		

^a^ Two different kinds of anions are present in the system. ^b^ Rate constants were expressed as the pseudo-first order rate parameters according to the observed rate law: *rate* = *k* [HCHO]_tot_ [H^+^] [Ph-NO], (Ph-NO = nitrosobenzene) and are an average of at least 3–4 runs. Standard errors are in parentheses. At 25 °C, in 92.6% acetonitrile–water, 0.100 M HClO_4_, and 0.200 M HCHO, except otherwise noted. ^c^ Primary kinetic isotope effect between formaldehyde and [D_2_]formaldehyde. ^d^ [HClO_4_] = 0.001 M. ^e^ HCHO]_tot_ = 0.259 M, [DCDO]_tot_ = 0.213 M. ^f^ From ref. [8]. ^g^ In 92.6% acetone–water. Relative to the extrapolated value for 92.6% acetone–water (see above). ^h^ In 80% acetone–water, [HClO_4_] = 0.050 M. ^i^ In 80% acetonitrile–water, [HClO_4_] = 0.050 M. ^j^ Rate constant for the reaction with 1.0 M acetaldehyde. Relative to the rate constant with [HClO_4_] = 0.100 M. See also Table 1.

**Table 3:** Properties of transition state structures in reaction step **2**→**3** obtained from ab initio calculations ^a^ in cases for proton transfer from nitrosocarbinolic cation C-H moiety to: water (**w-TS**), a chloride ion within the corresponding CIP (**TS-1**), water near a chloride ion within the corresponding SSIP (**TS-w**), a perchlorate ion within the corresponding CIP (**TS-p**), and water near a perchlorate ion within the corresponding SSIP (**TS-wp**).

	H Atom Acceptor	C⋯H/Å ^b^	C⋯H⋯A/° ^c^	*v*/cm^−1 d^	Δ*G*^‡^/kJ mol^−1 e^
**w-TS**	water	1.334	169.9	1468 *i*	+74.8
**TS-1**	Cl^−^	1.212	164.0	946 *i*	+24.6
**TS-w**	water⋯Cl^−^	1.312	171.9	1546 *i*	+56.0
**TS-p**	ClO_4_^−^	1.290	177.2	1601 *i*	+60.0
**TS-wp**	water⋯ClO_4_^−^	1.283	175.6	1400 *i*	+63.2

^a^ For details, please refer to the Supplementary Materials. ^b^ C-H distance. ^c^ C-H-Acceptor angle. ^d^ Normal mode imaginary frequency associated with the proton transfer. ^e^ Activation Gibbs energy calculated from the reactants, the nitrosocarbinolic-cation–anion ion pair, and the corresponding transition structure.

## Data Availability

Not applicable.

## Supplementary Materials

The following supporting information can be downloaded online: Figures S1–S6: Changes in the spectra, dependence of the *k*_obs_ on the volume fractions of acetonitrile and acetone, on the formaldehyde concentration, and on the Hammett σ constants for the reactions of formaldehyde with substituted nitrosobenzens; Figures S7–S18: Gibbs energy profiles and structures obtained from the ab initio calculations for ion pairs, transition structures, intermediates, and products involved in the reaction; Table S1: The influence of the cations on the observed rate constants in the reaction of formaldehyde and nitrosobenzenes; Table S2: The estimated values of the association constants for the acids and quaternary salts in acetonitrile–water; Tables S3–S5: The calculated PCM MP2 electronic and Gibbs formation energies and Cartesian coordinates for the structures involved in the reaction steps.
