# Supplementary material for: Intriguing Chloride: Involvement of Chloride Ions in Proton Transfers
Source: Molecules. 2022 Feb 18;27(4):1401. doi: 10.3390/molecules27041401 (PMC8880404; doi:10.3390/molecules27041401)
Supplement: Supplementary file 1 [file molecules-27-01401-s001.zip › molecules-1596308-supplementary.pdf]

Supplementary Materials:

Intriguing chloride; Involvement of Chloride Ions in Proton Transfers

by Viktor Pilepić *, Cvijeta Jakobušić Brala and Stanko Uršić *

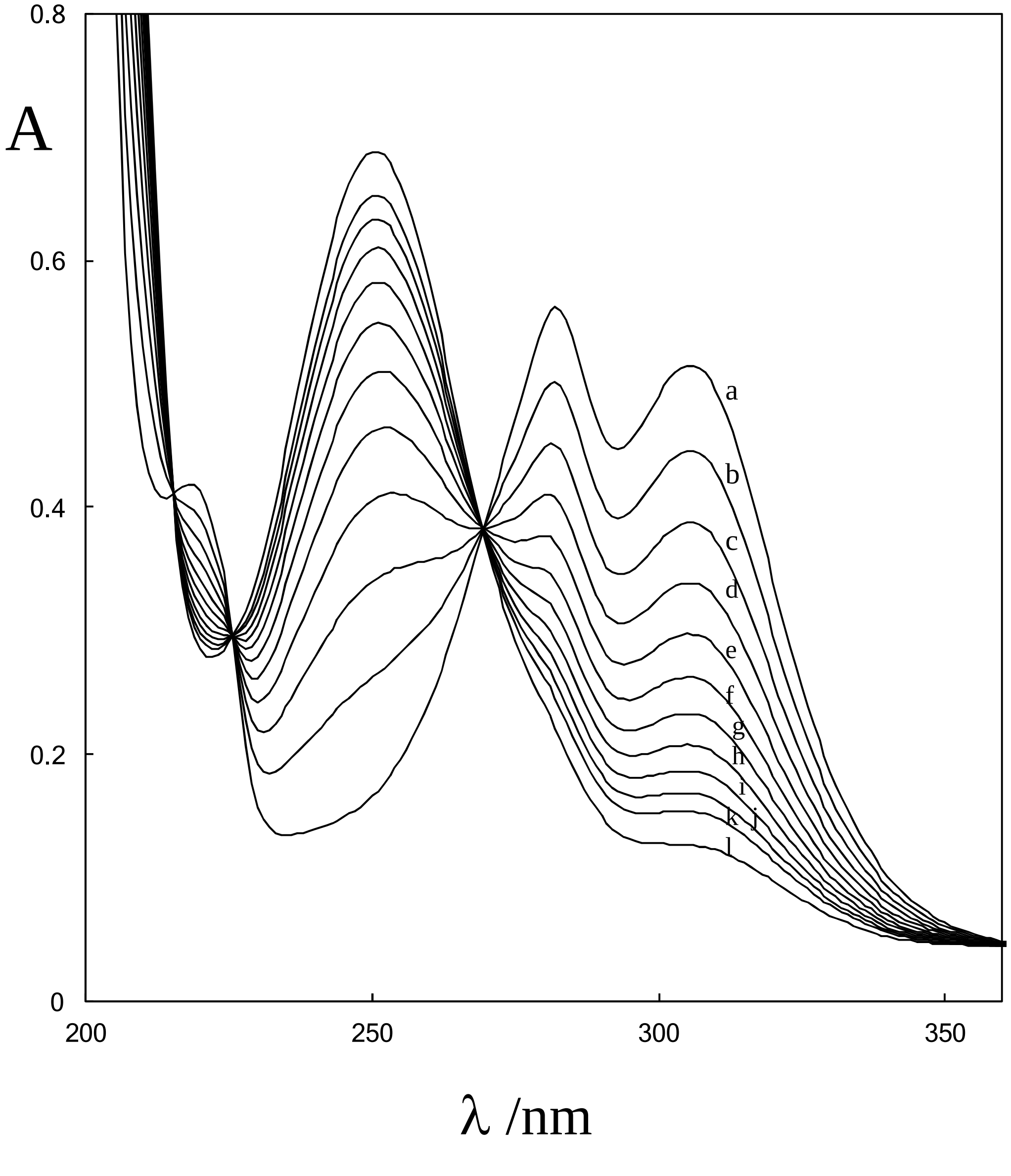

Figure S1. Change in the spectra of the reactants and products during the reaction of the formaldehyde with nitrosobenzene in 92.6 % acetonitrile-water solution. $[HCHO]_{tot}$ = 0.1 M, [Ph-NO] = 0.00005 M, [HCl] = 0.005 M. At 25°C. a - l the spectra recorded in 0, 90, 180, 270, 360, 450, 540, 630, 720, 810, 900 and 1116 seconds after mixing the reactants.

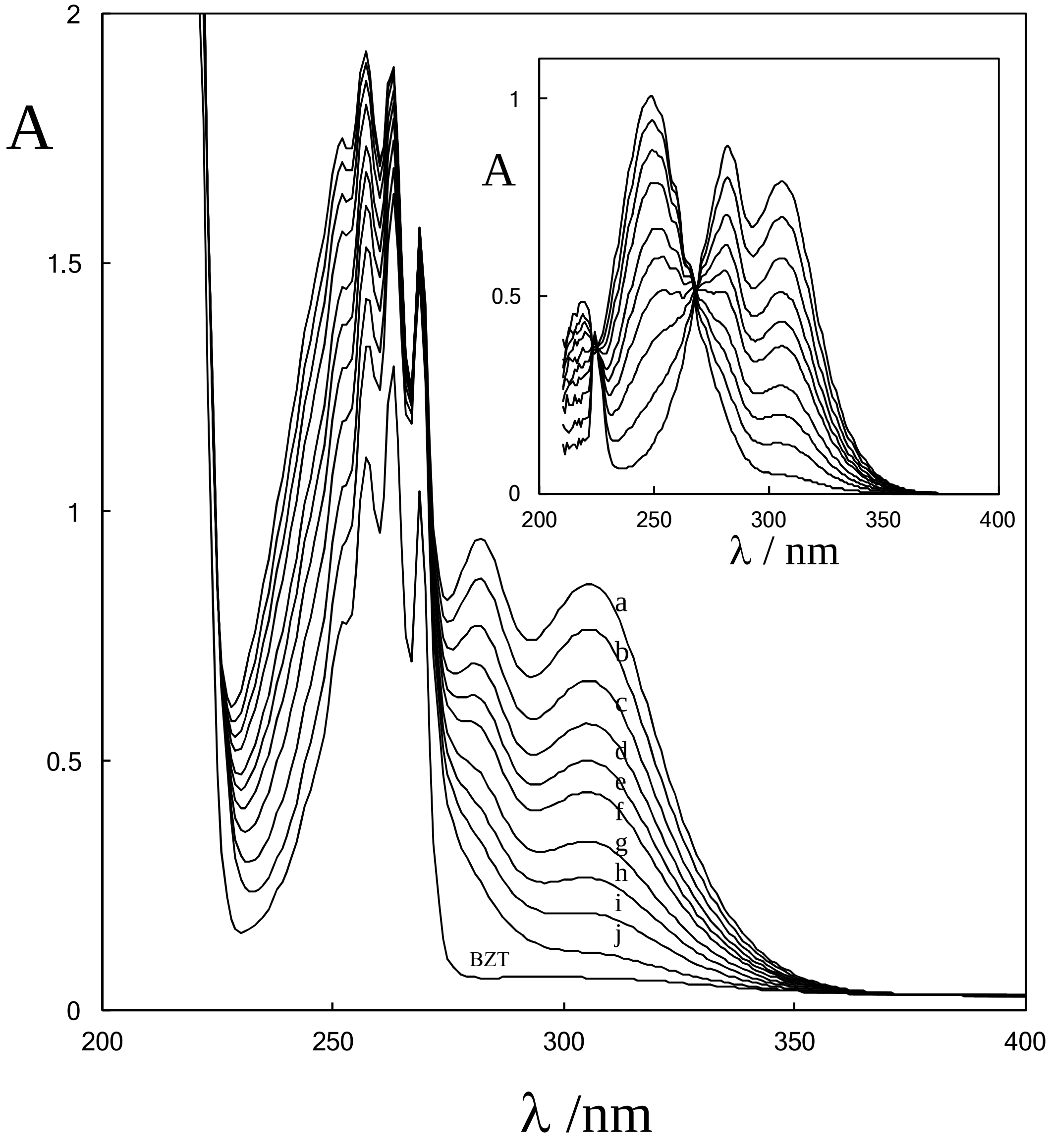

Figure S2. Change in the spectra of the reactants and products during the reaction of the formaldehyde with nitrosobenzene in 92.6 % acetonitrile-water solution of benzyltrimethylammonium perchlorate. $[HCHO]_{tot}$ = 0.2 M, $[HClO_4]$ = 0.1 M, [Ph-NO] = 0.0001 M, $[PhCH_2(CH_3)_2N^+ClO_4^-]$ = 0.00319 M. a - j the spectra recorded in 0, 72, 144, 216, 288, 360, 504, 648, 864 and 1296 seconds after mixing the reactants. BZT - the spectra of 0.00319 M $PhCH_2(CH_3)_2N^+ClO_4^-$ in the reaction solution.

Inset: Spectra were derived from the initial spectra by substraction the appropriate spectrum of $PhCH_2(CH_3)_2N^+ ClO_4^-$.

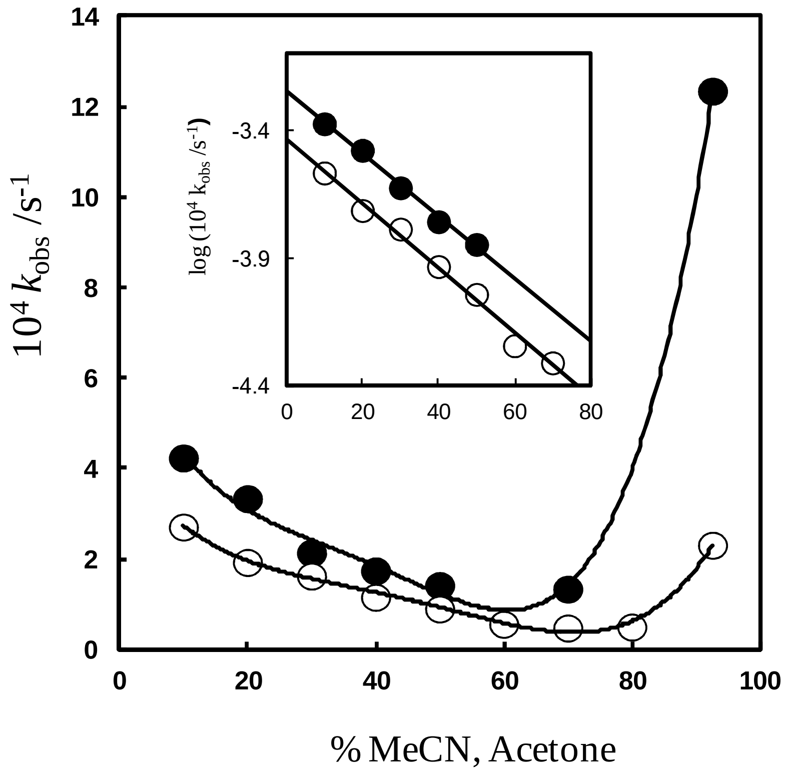

Figure S3. The dependence of the k_{obs} on the volume fractions of acetonitrile and acetone in the reaction of formaldehyde with nitrosobenzene. In water-acetonitrile (solid circles) and water- acetone (open circles) mixtures. At 25°. $[HCHO]_{tot}$ = 0.259 M, $[HClO_4]$ = 0.100 M. The points are average of 3-4 runs. Inset: the plot of the log k_{obs} on the volume fractions of acetonitrile and acetone in the above reaction.

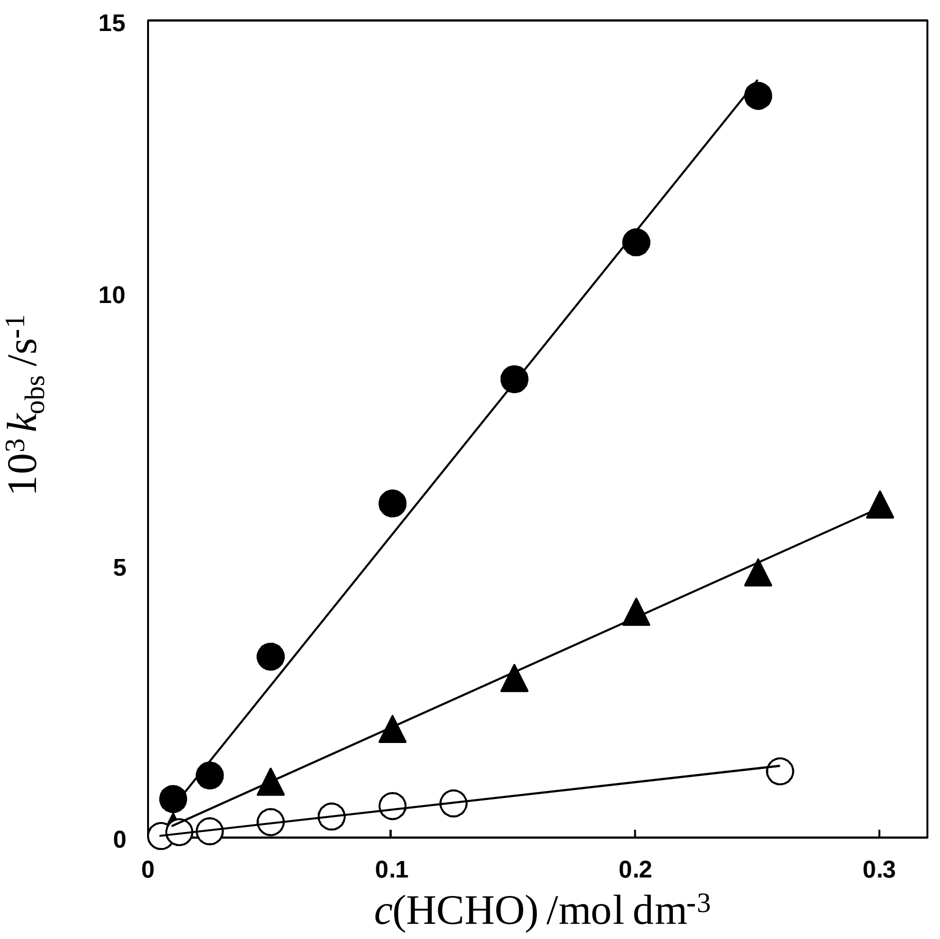

Figure S4. Dependence of the observed rate constants on the formaldehyde concentration for the reaction of nitrosobenzene with formaldehyde in 92.6 % acetonitrile-water solution in presence of $[PhCH_2(CH_3)_2N^+Cl^-]$ = 0.001 M, $[HClO_4]$ = 0.1 M (solid circles), [HCl] = 0.01 M (triangles) and $[HClO_4]$ = 0.1 M (open circles), at 25°C.

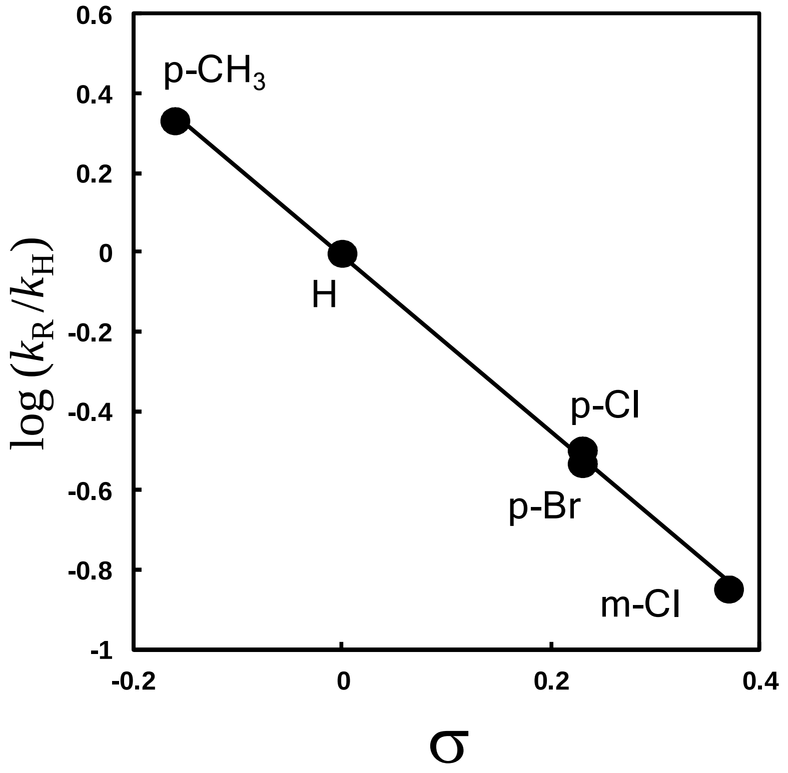

Figure S5. Dependence of the observed rate constants on the Hammett σ constants for the reaction of substituted nitrosobenzenes with formaldehyde in 92.6 % acetonitrile-water solution in presence of 0.0032 M of $PhCH_2(CH_3)_2N^+Cl^-$. $[HCHO]_{tot}$ = 0.330 M, $[HClO_4]$ = 0.100 M, at 25°C. (ρ = - 2.22, r = 0.9998)

Table S1. The influence of the cations on the observed rate constants in the reaction of formaldehyde and nitrosobenzenes.[a]

	Cations / Salt		$10^4\,k$ /s $^{-1}$
1	-	($HClO_4$ 0.100 M)	10.02 (0.01)
2	$(CH_3)_4N^+$	($(CH_3)_4N^+$ ClO_4^- 0.0032 M, $HClO_4$ 0.100 M)	14.06 (0.02)
3	$PhCH_2(CH_3)_3N^+$	($PhCH_2(CH_3)_3N^+$ ClO_4^- 0.0032 M, $HClO_4$ 0.100 M)	18.64 (0.69)
4	-	(HCl 0.005 M)	31.45 (0.25)
5	$(CH_3)_4N^+$	($(CH_3)_4N^+$ Cl^- 0.002 M, HCl 0.005 M)	36.22 (0.74)
6	$PhCH_2(CH_3)_3N^+$	($PhCH_2(CH_3)_3N^+$ Cl^- 0.002 M, HCl 0.005 M)	36.60 (0.67)

[a]At 25 °C, in 92.6 % acetonitrile-water solution. Rate constants expressed as described in Table 1. $[HCHO]_{tot}$= 0.200 M throughout.

Table S2. The estimated values of the association constants for the acids and quaternary salts in acetonitrile-water.

	Λ_o [a]	K / M^{-1}
HCl	133.6	200.5 (1.1)
HBr	142.9	63.9 (0.8)
H_2SO_4	110.8	154.6 (1.9)
$(CH_3)_4N^+$ Cl^-	130.0	31.3 (1.0)
$(CH_3)_4N^+$ ClO_4^-	192.6	101.5 (2.1)
$PhCH_2(CH_3)_3N^+$ Cl^-	142.4	87.5 (1.0)
$PhCH_2(CH_3)_3N^+$ Br^-	178.3	66.8 (0.8)
$PhCH_2(CH_3)_3N^+$ ClO_4^-	222.4	103.0 (4.0)
CCl_3COOH	>91 [b]	>7500 [b]
$MgCl_2$ [b, c]	179.9	206.7 (6.1)
$MgCl_2$ [b, d]	121.5	172.9 (2.1)

[a] Limiting molar conductivity (S cm^2 mol^{-1}). At 25°C, in 92.6 % acetonitrile-water. [b] Only crude estimation (r^2 = 0.9965-0.9988). [c] In 80 % acetonitrile-water, assuming arbitrarily that formation of $MgCl^+$ predominates in the case. [d] In 80 % acetone-water, the same assumptions as in c). (cf. Table 1, entries 5 and 8).

Results of the *ab initio* molecular orbital calculations.

In effort to shed light on the ion pairs and specific role of chloride ion in the proton transfer from carbon, molecular orbital calculations for the proposed species involved in the reaction steps were performed. All computations were performed using GAUSSIAN 09 Rev. D.01 package [48] at MP2/6-311++G(d,p) level with tight or very tight convergence thresholds. Non-specific solvent effects have been estimated by using polarizable continuum model (PCM) of self-consistent reaction field (SCRF) method [49] with acetonitrile as a solvent. The obtained stationary points were confirmed either as minima or saddle points by vibrational analysis at the same theory level. The geometry optimizations have been started from numerous different initial structures and also the "chemically intuitive" different positions of the chloride or perchlorate anion in the nitrosocarbinolic cation - anion contact ion pair (**CIP**) and transition structures for the H atom transfer to anion. For a series of the structures obtained, those with the lowest energy (and thus the most populated) or the representative are shown for clarity. In the cases of the nitrosocarbinolic cation - anion solvent separated ion pair (**SSIP**) and the corresponding transition structures for the H atom transfer from C atom to a water molecule located between the anion and nitrosocarbinolic cation, the geometry optimizations have been started also from numerous different initial structures and the "chemically intuitive" positions of water molecule and chloride or perchlorate anion. No stable structure with the water molecule between nitrosocarbinolic cation and anion as would be expected for solvent-separated or solvent-shared ion pairs could be found, and the geometry optimizations mostly collapsed in the structures that can be described as a "solvent-shared like" ion pair which are shown and discussed further.

The pictorial representation of the noncolvalent interaction (NCI) plots from the obtained electron densities of the nitrosocarbinolic cation - chloride contact ion pair **2** were performed with the MULTIWFN [50] and VMD [51] software packages. The NCI plots were obtained by plotting the sign(λ_2)$\rho(r)$ values with two cutoff values, ρ^{+}_{cut} and ρ^{-}_{cut}, color-mapped onto the reduced density gradient (RDG) isosurface, $s(r)$. The density cutoff values were used for setting the color scale of the obtained domains, red-green-blue for ρ^{+}_{cut} (red) and ρ^{-}_{cut} (blue), respectively.

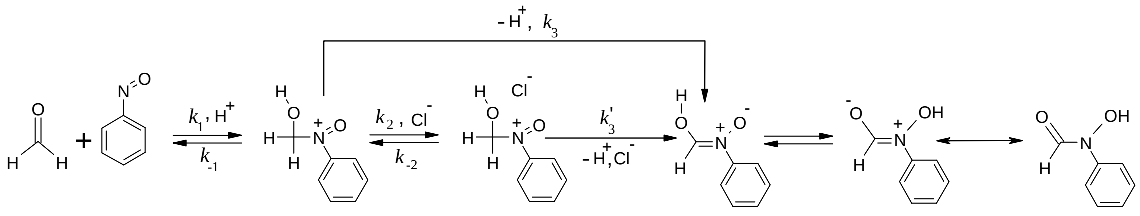

1 2 3 4

Scheme 1.

Structures in the **1→2→3** reaction steps in presence of chloride ion.

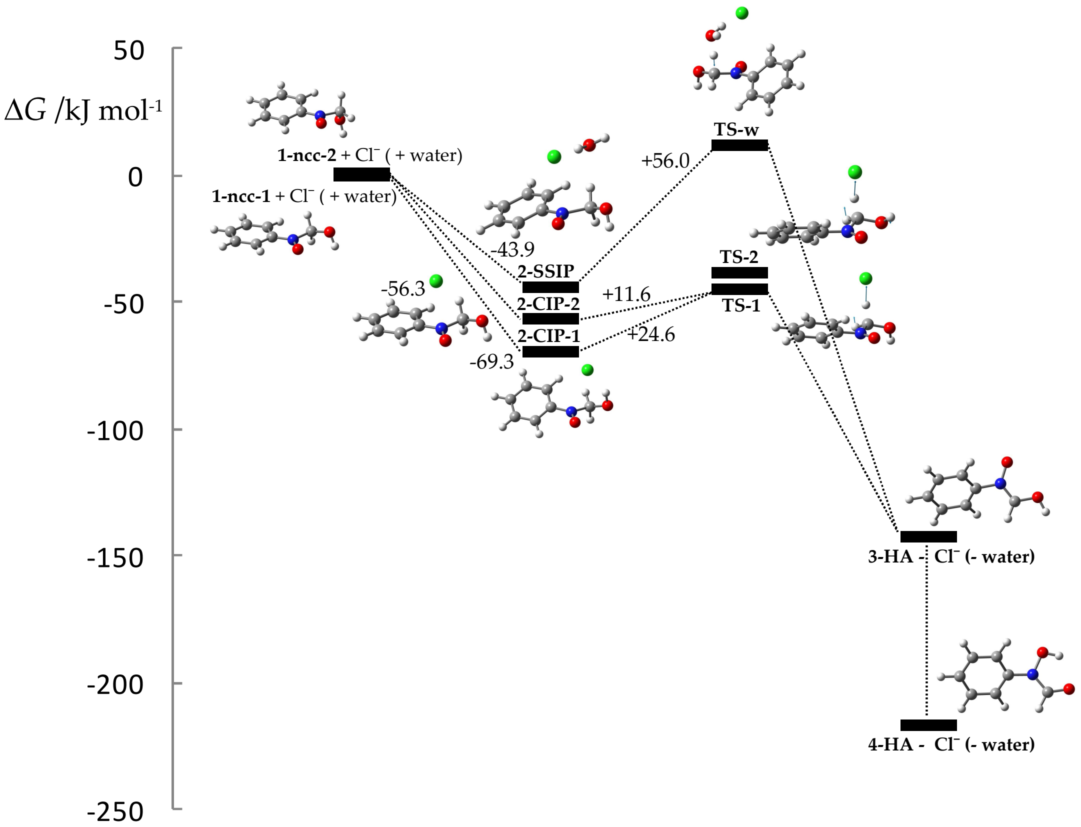

Figure S6. Gibbs energy profile for the **1→2→3** reaction steps (Scheme 1) from nitrosocarbinolic cation intermediate **1 (1-ncc-1** and **1-ncc-2)** and chloride ion (and water) for the reaction of nitrosobenzene with formaldehyde in presence of the chloride ions. Species involved in reaction steps: **2-CIP-1** and **2-CIP-2** (nitrosocarbinolic cation - chloride contact ion pair **2** conformers in **Scheme 1**); **2-SSIP** (nitrosocarbinolic cation - chloride solvent "shared-like" ion pair); **TS-1** and **TS-2** (transition structures for the proposed proton transfer from nitrosocarbinolic cation C atom to chloride ion within nitrosocarbinolic cation - chloride contact ion pair in the reaction step **2→3**); **TS-w** (transition structure for proton transfer from nitrosocarbinolic cation C atom to a nearby water molecule within nitrosocarbinolic cation - chloride solvent "shared-like" ion pair); **3-HA** and **4-HA** (forms of the product, hydroxamic acid, **3** and **4** in **Scheme 1**). The ΔG for the formation of nitrosocarbinolic cation intermediates **1-ncc-1** and **1-ncc-2** from the reactants (nitrosobenzene, formaldehyde and hydronium ion) are −77.2 kJ mol^{-1} and −76.1 kJ mol^{-1}, respectively. Data from **Table S3**.

Table S3. The PCM MP2 Electronic and Gibbs energies for calculated structures in the **1**→**2**→**3** reaction steps in presence of chloride ion. The nitrosocarbinolic cation intermediate **1** conformers (**1-ncc-1** and **1-ncc-2**), nitrosocarbinolic cation - chloride contact ion pair **2** (**2-CIP-1** and **2-CIP-2**), nitrosocarbinolic cation - chloride "solvent-shared like" ion pair (**2-SSIP**), transition structures for the proposed proton transfer from nitrosocarbinolic cation C atom to chloride ion within nitrosocarbinolic cation - chloride contact ion pair (**TS-1** and **TS-2**), transition structures for proton transfer from nitrosocarbinolic cation C atom to a water molecule within nitrosocarbinolic cation - chloride "solvent-shared like" ion pair (**TS-w**) and forms of the product, hydroxamic acid (**3-HA** and **4-HA**, **3** and **4** in **Scheme 1**) calculated at MP2/6-311++G(d,p) level by using PCM-SCRF method with acetonitrile as a solvent.

structure	E(MP2) /Hartree	ΔG(MP2) /Hartree	ΔG / kJ mol^{-1} [a]
**1-ncc-1**	-475.311265	-475.202447	0.0
**1-ncc-2**	-475.310973	-475.202019	+1.1
**2-CIP-1**	-935.148560	-935.042011	-69.3
**2-CIP-2**	-935.141065	-935.037072	-56.3
**2-SSIP**	-1011.435622	-1011.311177	-43.9
**TS-1**	-935.133045	-935.032648	-44.7
**TS-2**	-935.129651	-935.030162	-38.1
**TS-w**	-1011.411430	-1011.289845	12.1
**3-HA**	-474.907043	-474.810803	-142.3
**4-HA**	-474.934655	-474.839118	-216.7
water	-76.282756	-76.278821	
Cl^-	-459.813188	-459.813188	
H_3O^+	-76.666993	-76.652581	

[a] The ΔG for the formation from **1-ncc-1**, chloride ion and water. The ΔG for the formation of nitrosocarbinolic cation intermediates **1-ncc-1** and **1-ncc-2** from the reactants (nitrosobenzene, formaldehyde and hydronium ion) are –77.2 kJ mol^{-1} and –76.1 kJ mol^{-1}, respectively.

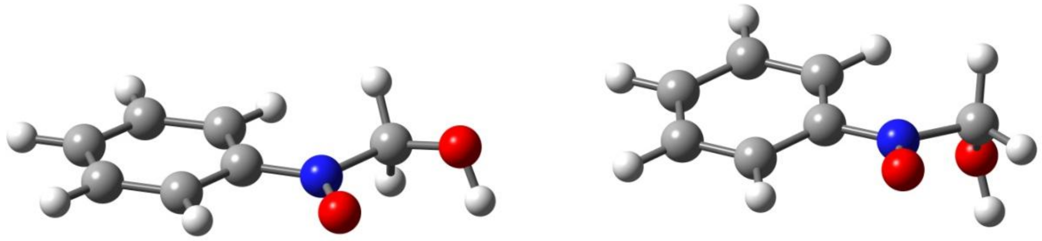

1-ncc-1 **1-ncc-2**

Figure S7. Optimized stuctures of the nitrosocarbinolic carbocation intermediate **1** conformers (**1-ncc-1** and **1-ncc-2**). The ΔG for formation of the nitrosocarbinolic cation intermediates from the reactants (nitrosobenzene, formaldehyde and hydronium ion) are –77.2 kJ mol^{-1} and –76.1 kJ mol^{-1}, respectively.

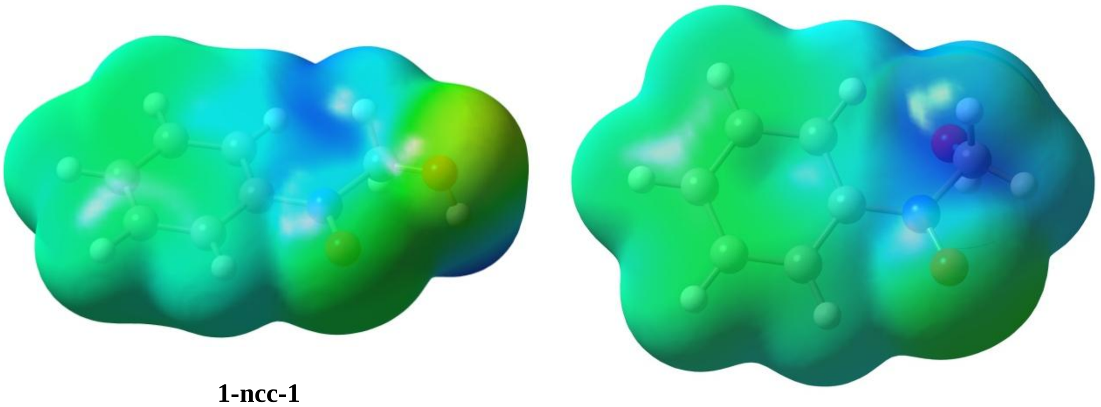

1-ncc-1 **1-ncc-2**

Figure S8. Molecular electrostatic potential (MEP) plotted on the 0.001 a.u. electron density isosurface of the optimized structure of the nitrosocarbinolic cation intermediate **1** conformers (**1-ncc-1** and **1-ncc-2**). The more positive region of the MEP is shown in blue color.

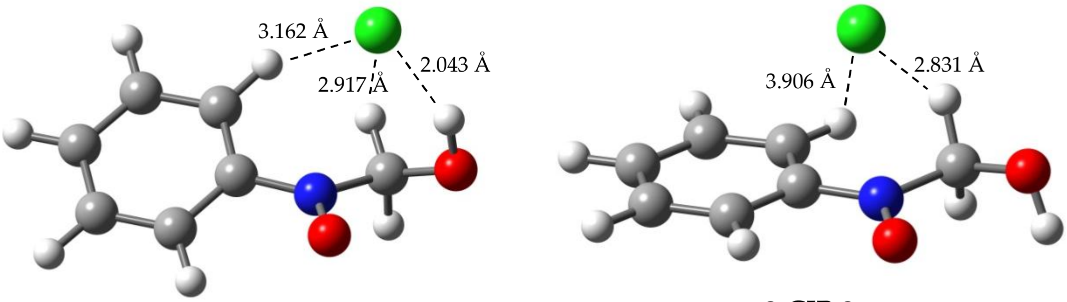

2-CIP-2

2-CIP-1

Figure S9. The lowest energy and representative stuctures of the nitrosocarbinolic cation - chloride contact ion pair **2**. The Δ*G* for the formation of **2-CIP-1** and **2-CIP-2** from **1-ncc-1** and chloride ion are –69.3 kJ mol^{-1} and –56.3 kJ mol^{-1}, respectively.

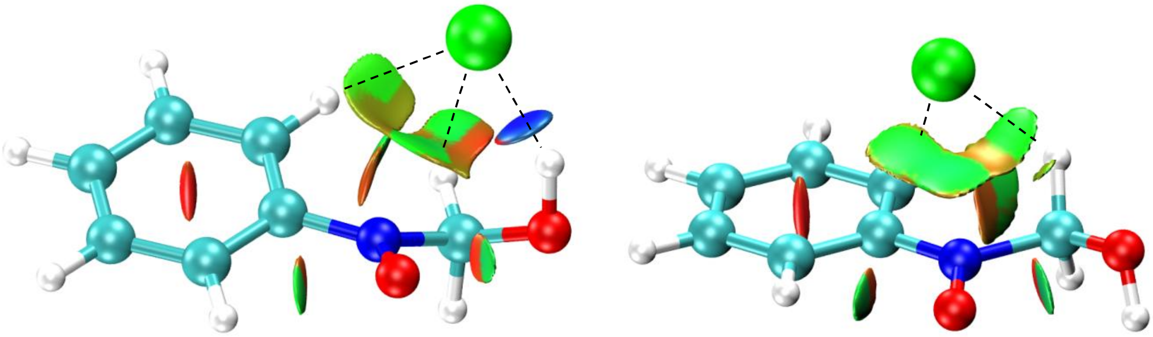

2-CIP-2

2-CIP-1

Figure S10 Selected non-covalent interaction (NCI) domains in the nitrosocarbinolic cation - chloride contact ion pair **2** structures. The reduced gradient isosurfaces at *s* = 0.5 a.u. are colored on a red-green-blue color scale according to the sign(λ_2)ρ values ranging from -0.05 to 0.05 a.u. indicating repulsive non-bonding, weak attractive and strong attractive interactions, respectively.

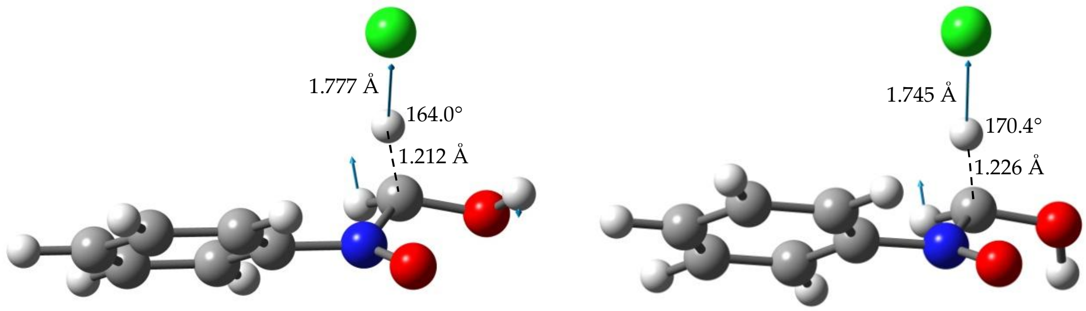

TS-1 **TS-2**

Figure S11. Transition structures for the proposed proton transfer from nitrosocarbinolic cation C atom to chloride ion within nitrosocarbinolic cation - chloride contact ion pair in reaction step **2**→**3**. The $\Delta G^‡$ for **TS-1** and **TS-2** from **2-CIP-1** are +24.6 kJ mol^{-1} and +31.2 kJ mol^{-1}, respectively. Normal mode displacement vectors shown (blue arrows) for unique imaginary frequency of 946i cm^{-1} and 1009i cm^{-1} for transition structures **TS-1** and **TS-2** respectively are associated primarily with a motion of the H atom between C atom of the nitrosocarbinolic cation and chloride ion and the second H atom on this C atom.

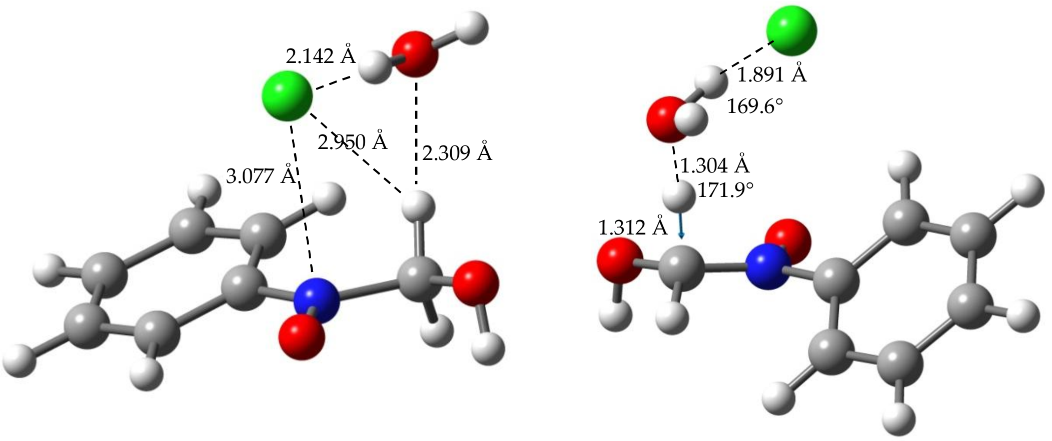

2-SSIP **TS-w**

Figure S12. Optimized stuctures of the nitrosocarbinolic cation - chloride "solvent shared-separated-like" ion pair (**2-SSIP**) and transition structure (**TS-w**) for proton transfer from nitrosocarbinolic cation C atom to water molecule in a close presence of chloride ion within nitrosocarbinolic cation - chloride "solvent shared-separated-like" ion pair in a reaction step **2**→**3**. The ΔG for **2-SSIP** formation from nitrosocarbinolic cation, chloride ion and water molecule is –43.9 kJmol^{-1} and $\Delta G^‡$ for **TS-w** from **2-SSIP** is +56.0 kJmol^{-1}. Normal mode displacement vector shown (blue arrow) for unique imaginary frequency of 1546i cm^{-1} for **TS-w** is associated with a motion of the H atom between C atom of the nitrosocarbinolic cation and O atom of the water molecule.

Structures in the corresponding **1**→**2**→**3** reaction steps in presence of perchlorate ion.

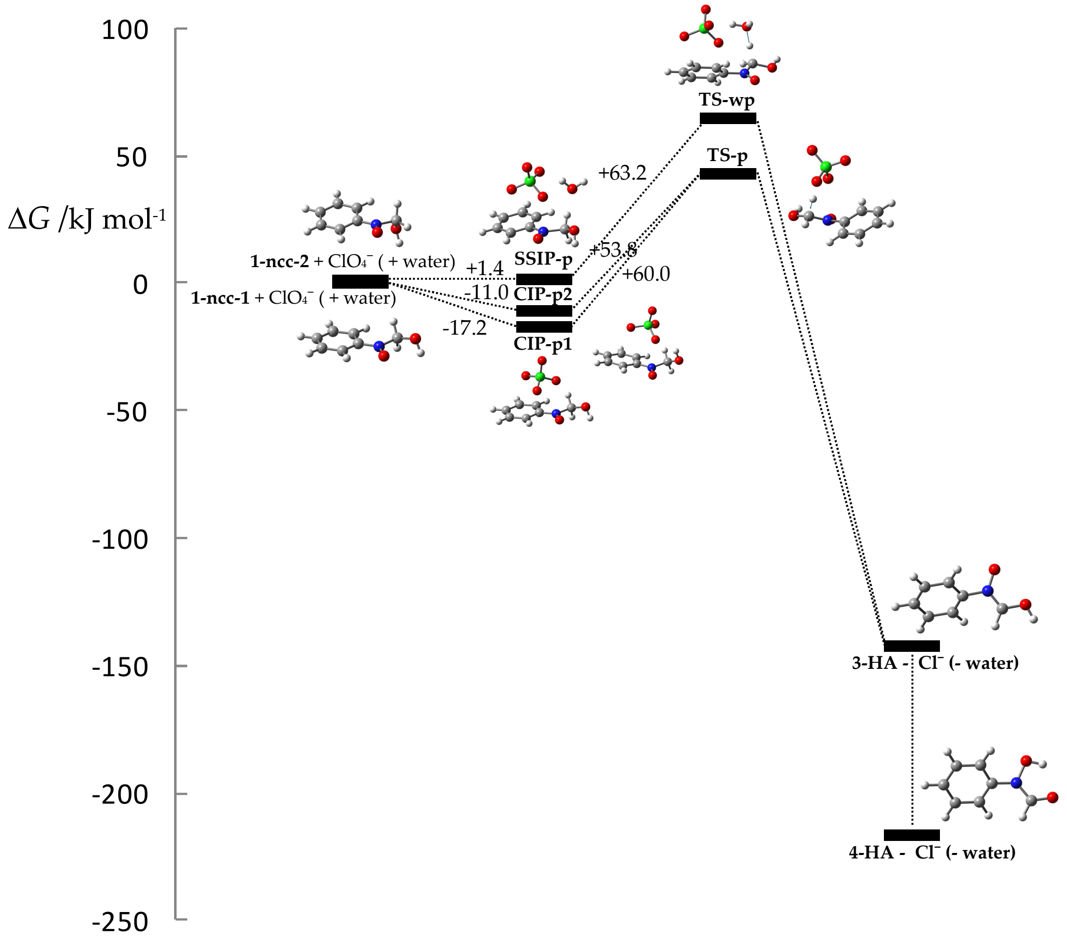

Figure S13. Gibbs energy profile for the corresponding **1**→**2**→**3** reaction steps (Scheme 1) from nitrosocarbinolic cation intermediate **1 (1-ncc-1** and **1-ncc-2)** and perchlorate ion (and water) for the reaction of nitrosobenzene with formaldehyde in presence of the perchlorate ions. Species involved in the reaction steps: **CIP-p1** and **CIP-p2** (nitrosocarbinolic cation - perchlorate contact ion pairs); **SSIP-p** (nitrosocarbinolic cation - perchlorate solvent "shared-like" ion pair); **TS-p** (transition structure for proton transfer from nitrosocarbinolic cation C atom to perchlorate ion within nitrosocarbinolic cation - perchlorate contact ion pair in the reaction step **2**→**3**); **TS-wp** (transition structure for proton transfer from nitrosocarbinolic cation C atom to a nearby water molecule within nitrosocarbinolic cation - perchlorate solvent "shared-like" ion pair); **3-HA** and **4-HA** (forms of the product, hydroxamic acid, **3** and **4** in **Scheme 1**). Data from **Table S4**.

Table S4. The calculated PCM MP2 Electronic and Gibbs energies for calculated structures in the **1→2→3** reaction steps in presence of perchlorate ion. The nitrosocarbinolic cation intermediate **1** conformers (**1-ncc-1** and **1-ncc-2**), nitrosocarbinolic cation - perchlorate contact ion pair (**CIP-p1** and **CIP-p2**), nitrosocarbinolic cation - perchlorate "solvent-shared like" ion pair (**SSIP-p**), transition structure for the proton transfer from nitrosocarbinolic cation C atom to perchlorate ion within nitrosocarbinolic cation - perchlorate contact ion pair (**TS-p**), transition structure for proton transfer from nitrosocarbinolic cation C atom to a water molecule within nitrosocarbinolic cation - perchlorate "solvent-shared like" ion pair (**TS-wp**) and forms of the product, hydroxamic acid (**3-HA** and **4-HA**, **3** and **4** in **Scheme 1**) calculated at MP2/6-311++G(d,p) level by using PCM-SCRF method with acetonitrile as a solvent.

structure	E(MP2) /Hartree	ΔG(MP2) /Hartree	ΔG / kJ mol^{-1}
**1-ncc-1**	-475.311265	-475.202447	0.0
**1-ncc-2**	-475.310973	-475.202019	+1.1
**CIP-p1**	-1235.084743	-1234.967494	-17.2
**CIP-p2**	-1235.080486	-1234.965127	-11.0
**TS-p**	-1235.055751	-1234.944625	42.9
**SSIP-p**	-1311.375799	-1311.239239	1.4
**TS-wp**	-1311.346887	-1311.215161	64.6
**3-HA**	-474.907043	-474.810803	-142.3
**4-HA**	-474.934655	-474.839118	-216.7
water	-76.282756	-76.278821	
H_3O^+	-76.666993	-76.652581	
ClO_4^-	-759.748716	-759.758505	

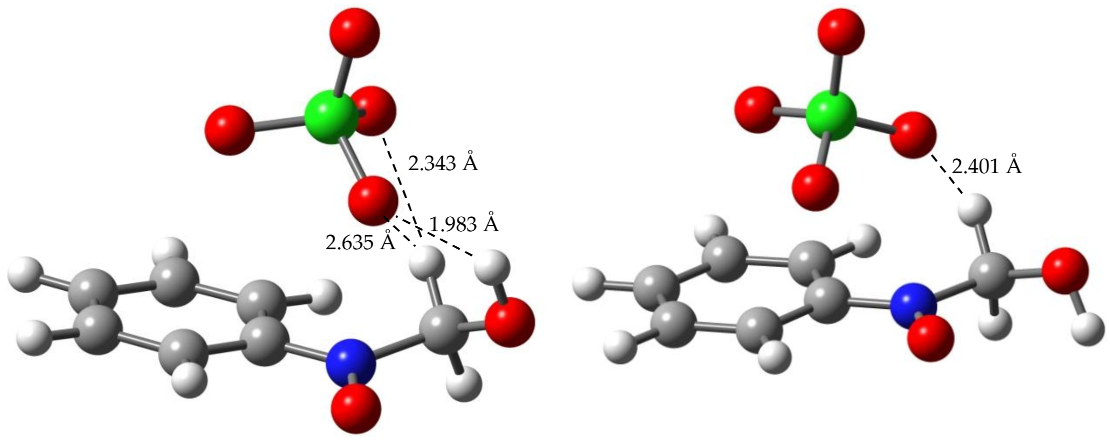

CIP-p1 **CIP-p2**

Figure S14. The representative optimized stuctures of the nitrosocarbinolic cation - perchlorate contact ion pair. The Δ*G* for the formation of **CIP-p1** and **CIP-p2** from **1-ncc-1** and perchlorate ion are –17.2 kJ mol^{-1} and –11.0 kJ mol^{-1}, respectively.

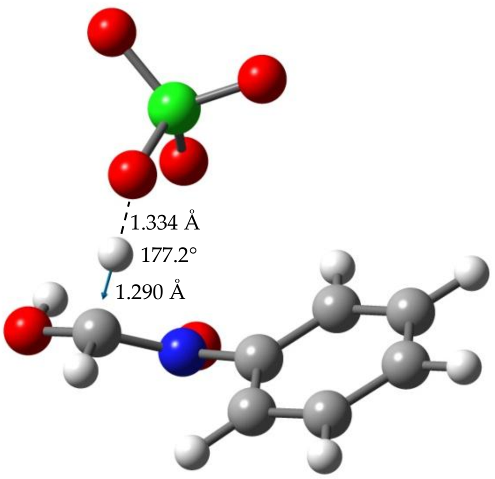

TS-p

Figure S15. Transition structure for the proposed proton transfer from nitrosocarbinolic cation C atom to perchlorate ion within nitrosocarbinolic cation - perchlorate contact ion pair in reaction step **2**→**3**. The $\Delta G^{\ddagger}$ for **TS-p** from **CIP-p1** is +60.0 kJ mol^{-1}. Normal mode displacement vector shown (blue arrows) for unique imaginary frequency of 1601i cm^{-1} is associated primarily with a motion of the H atom between C atom of the nitrosocarbinolic cation and perchlorate ion.

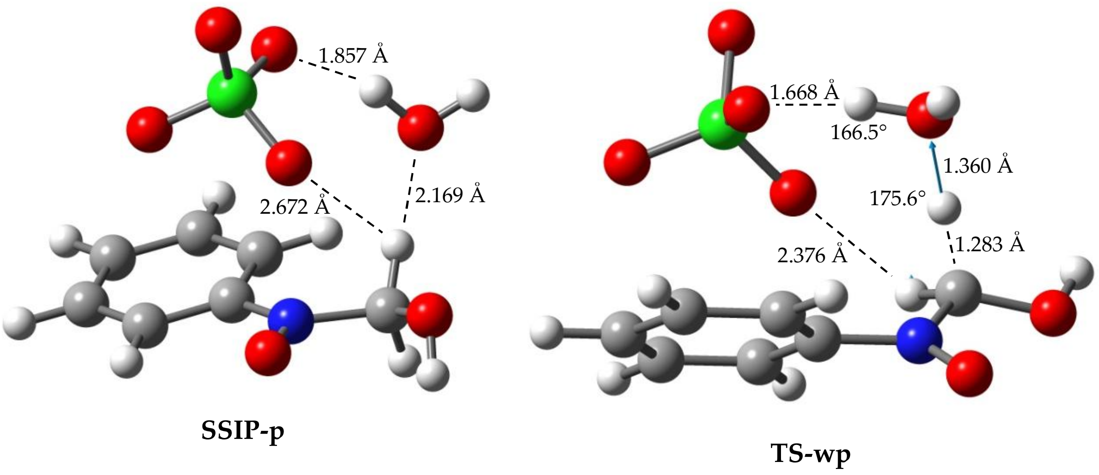

Figure S16. Optimized stuctures of the nitrosocarbinolic cation - perchlorate "solvent shared-separated-like" ion pair (**SSIP-p**) and transition structure (**TS-wp**) for proton transfer from nitrosocarbinolic cation C atom to water molecule in a close presence of perchlorate ion within nitrosocarbinolic cation - perchlorate "solvent shared-separated-like" ion pair in a reaction step **2**→**3**. The Δ*G* for **SSIP-p** formation from nitrosocarbinolic cation, perchlorate ion and water molecule is +1.4 kJmol^{-1} and Δ*G*‡ for **w-TS** from **SSIP-p** is +63.2 kJmol^{-1}. Normal mode displacement vector shown (blue arrow) for unique imaginary frequency of 1400i cm^{-1} for **TS-wp** are associated primarily with a motion of the H atom between C atom of the nitrosocarbinolic cation and O atom of the water molecule.

Proton transfer to water.

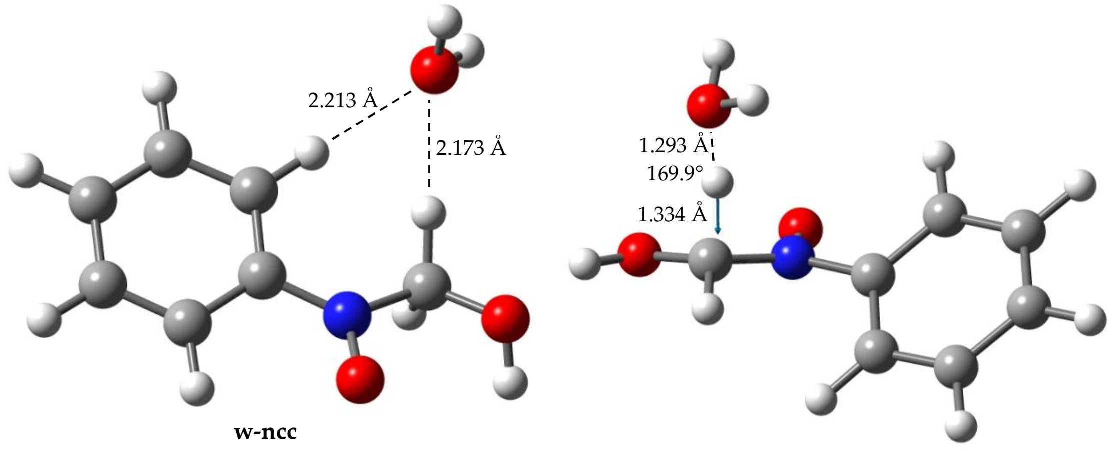

w-ncc

w-TS

Figure S17. Optimized stuctures of the nitrosocarbinolic cation - water adduct (**w-ncc**) and transition structure for the proton transfer from nitrosocarbinolic cation C atom to water molecule (**w-TS**). Normal mode displacement vector shown (blue arrow) for unique imaginary frequency of 1468i cm^{-1} for transition structure **w-TS** are associated primarily with a motion of the H atom between C atom of the nitrosocarbinolic cation and O atom of the water molecule.

Table S5. The calculated PCM MP2 Electronic and Gibbs energies for the nitrosocarbinolic cation - water adduct (**w-ncc**) and transition structure for the proton transfer from nitrosocarbinolic cation C atom to water molecule (**w-TS**). calculated at MP2/6-311++G(d,p) level by using PCM-SCRF method with acetonitrile as a solvent.

structure	*E*(MP2) /Hartree	Δ*G*(MP2) /Hartree	Δ*G* / kJ mol^{-1}
**w-ncc**	-551.603584	-551.477045	+11.0[a]
**w-TS**	-551.571402	-551.446276	+74.8[b]

[a] The Δ*G* for the **w-ncc** formation from nitrosocarbinolic cation and water.

[b] The $\Delta G^{\ddagger}$ for the proton transfer from C atom of nitrosocarbinolic cation to water molecule.

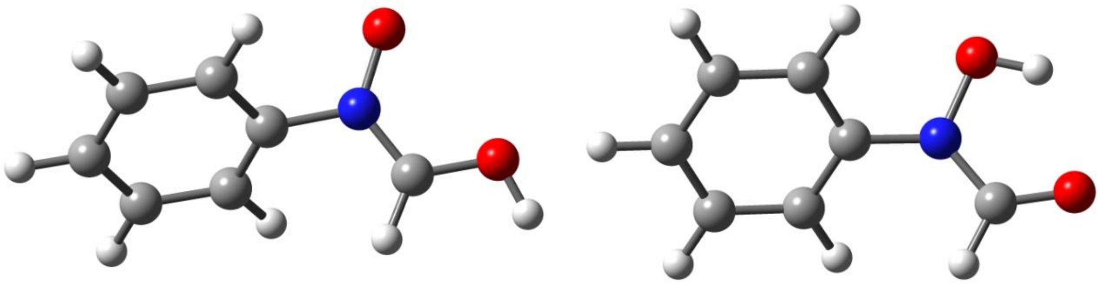

3-HA

4-HA

Figure S18. Optimized stuctures of the products **3-HA** and **4-HA** hydroxamic acid.

Cartesian coordinates of the optimized structures at MP2/6-311++G(d,p) level by using solvent polarizable continuum model (PCM) of self-consistent reaction field (SCRF) method with acetonitrile as a solvent.

Nitrosocarbinolic cation intermediate **1** (**1-ncc-1**):

C	2.139646	-0.837700	0.026431
H	1.820559	-1.477879	-0.793625
H	2.013878	-1.317615	1.000742
O	3.391471	-0.366778	-0.231129
N	1.134921	0.337150	0.048344
H	3.760936	0.030480	0.566807
O	1.614354	1.459765	0.106824
C	-0.246805	0.123518	0.016267
C	-0.743290	-1.199146	0.044223
C	-1.095761	1.252606	-0.035035
C	-2.466457	1.039783	-0.060044
C	-2.123034	-1.378083	0.028220
H	-0.671586	2.250189	-0.062737
H	-0.088255	-2.060721	0.093038
C	-2.980785	-0.269335	-0.028221
H	-3.140807	1.888179	-0.107061
H	-2.531357	-2.382472	0.055334
H	-4.055492	-0.423970	-0.047518

Nitrosocarbinolic cation intermediate **1** (**1-ncc-2**):

C	2.353905	-0.420486	-0.271307
H	3.255867	0.190622	-0.226748
H	2.252724	-0.915559	-1.237638
O	2.212357	-1.337585	0.742541
N	1.234260	0.627009	-0.221159
H	2.584865	-0.968679	1.553388
O	1.584141	1.804897	-0.261337
C	-0.105482	0.260909	-0.106691
C	-0.479063	-1.079490	-0.360978
C	-1.043654	1.261111	0.242585
C	-2.379783	0.901118	0.325960
C	-1.830450	-1.400590	-0.293580
H	-0.706612	2.269798	0.456244
H	0.250117	-1.830085	-0.638096
C	-2.774463	-0.423711	0.058012
H	-3.120605	1.641824	0.607549
H	-2.149521	-2.415833	-0.503400
H	-3.824702	-0.692813	0.123172

Nitrosocarbinolic cation (**1**) - chloride contact ion pair **2** (**2-CIP-1**):

C	-1.487891	-0.706405	1.227086
H	-1.261918	0.282978	1.619796
H	-1.180868	-1.491705	1.923700
O	-2.772503	-0.883812	0.825829
N	-0.529259	-0.906995	0.040170
H	-2.950283	-0.093478	0.257213
O	-0.872423	-1.692383	-0.828561
C	0.760121	-0.349382	0.039910
C	1.005346	0.869557	0.705348
C	1.777989	-1.078549	-0.611180
C	3.072439	-0.575142	-0.573183
C	2.311561	1.352794	0.714911
H	1.547153	-2.030773	-1.077345
H	0.200886	1.456135	1.129964
C	3.339952	0.638624	0.082935
H	3.877628	-1.127785	-1.046071
H	2.524406	2.300689	1.198001
H	4.354879	1.024412	0.106153
Cl	-2.289693	1.513475	-0.816806

Nitrosocarbinolic cation (**1**) - chloride contact ion pair **2** (**2-CIP-2**):

C	-1.806528	-1.046032	0.720342
H	-1.640312	-0.296885	1.489451
H	-1.489960	-2.049316	1.022284
O	-3.095308	-0.963861	0.278925
N	-0.839191	-0.678365	-0.417945
H	-3.300793	-1.735337	-0.262639
O	-1.299517	-0.679151	-1.546738
C	0.520949	-0.443446	-0.176745
C	1.031034	-0.617913	1.125105
C	1.333126	-0.059914	-1.263065
C	2.687467	0.139343	-1.031837
C	2.394727	-0.421691	1.325244
H	0.894506	0.079312	-2.244757
H	0.400868	-0.913591	1.954985

C	3.218644	-0.033675	0.258627
H	3.332816	0.443617	-1.849366
H	2.813685	-0.557884	2.316648
H	4.278896	0.127768	0.429895
Cl	-1.207840	2.217350	0.262682

Nitrosocarbinolic cation (**1**) - chloride solvent shared ion pair (**2-SSIP**):

C	-0.845684	0.499004	0.145090
C	-1.916847	1.131709	-0.518991
C	-0.994203	-0.727394	0.826531
N	0.379703	1.178714	0.205156
C	-2.254687	-1.318152	0.835857
C	-3.157222	0.506544	-0.509683
H	-1.762749	2.086584	-1.009673
H	-0.160840	-1.228157	1.304753
O	0.600880	2.168774	-0.470404
C	1.445007	0.774742	1.231993
C	-3.331038	-0.709814	0.173140
H	-3.998129	0.974738	-1.010695
H	-2.394763	-2.264173	1.348316
H	0.980039	1.003475	2.195976
O	2.616305	1.427451	0.982150
H	-4.307545	-1.185124	0.184724
H	2.552212	2.336805	1.297657
H	1.580208	-0.292432	1.081035
Cl	1.524833	-0.837363	-1.817752
O	1.867559	-2.560568	0.760196
H	1.760148	-2.020514	-0.047735
H	2.821425	-2.652126	0.842182

Transition structure for proton transfer to chloride within **1**,chloride CIP (**TS-1**):

C	-0.730243	-0.415368	0.238453
C	-1.283581	0.612834	1.016876
C	-1.431717	-1.051175	-0.797495
N	0.602375	-0.815750	0.531652
C	-2.743451	-0.640860	-1.043373
C	-2.588541	1.013746	0.737495
H	-0.687933	1.088215	1.789162
H	-1.000257	-1.880634	-1.347134
O	1.037293	-0.711827	1.689602
C	1.531700	-0.995421	-0.530998
C	-3.317072	0.391117	-0.288310
H	-3.035513	1.819782	1.310822
H	-3.317463	-1.131842	-1.822666
H	1.075585	-1.452987	-1.406135
O	2.698296	-1.605457	-0.160932
H	-4.334649	0.708660	-0.495973
H	3.148358	-1.039167	0.481344
H	1.696599	0.187430	-0.736529
Cl	2.101845	1.909402	-0.571748

Transition structure for proton transfer to chloride within **1**,chloride CIP (**TS-2**):

C	-0.680813	-0.472951	0.227044
C	-1.272347	0.465465	1.085572
C	-1.346825	-1.019083	-0.880192
N	0.659086	-0.870548	0.515056
C	-2.659811	-0.607200	-1.120193
C	-2.576133	0.874091	0.810394
H	-0.704504	0.874753	1.914597
H	-0.889998	-1.788639	-1.493062
O	1.041068	-0.908302	1.693023
C	1.600525	-0.882079	-0.550254
C	-3.268875	0.341282	-0.287918
H	-3.050402	1.615109	1.446203
H	-3.206532	-1.032425	-1.955817
H	1.147182	-1.231299	-1.478483
O	2.828142	-1.381513	-0.211293
H	-4.285904	0.663020	-0.491759
H	2.883083	-2.301248	-0.500304
H	1.700185	0.336570	-0.635475
Cl	1.886193	2.063492	-0.468933

Transition structure for proton transfer to water within **1**,chloride SSIP (**TS-w**):

C	0.960209	0.386037	0.321925
C	1.157026	-0.972658	0.597435
C	1.944012	1.203439	-0.248464
N	-0.315861	0.945596	0.680472
C	3.175724	0.618615	-0.559038
C	2.392145	-1.531499	0.266512
H	0.340215	-1.560397	1.003190
H	1.783758	2.266429	-0.394370
O	-0.852430	0.578765	1.733832
C	-0.992233	1.728636	-0.263860

C	3.399058	-0.741246	-0.307817
H	2.567559	-2.586882	0.451446
H	3.963678	1.232780	-0.983758
H	-0.295716	2.340990	-0.842821
O	-2.150929	2.307646	0.178786
H	4.358339	-1.185245	-0.557078
H	-1.937251	3.059755	0.748703
H	-1.343377	0.809955	-1.119488
Cl	-2.189573	-2.130726	0.116005
O	-1.867956	-0.093303	-1.914168
H	-2.019549	-0.878476	-1.290684
H	-1.229031	-0.388563	-2.578275

Nitrosocarbinolic cation (**1**) - perchlorate contact ion pair (**CIP-p1**):

C	0.569283	2.151078	0.916721
H	0.735410	1.231967	1.475835
H	0.046173	2.913349	1.497815
O	1.700522	2.674969	0.361126
N	-0.414774	1.757980	-0.209117
H	2.171432	1.919445	-0.029038
O	-0.308021	2.352279	-1.270796
C	-1.350267	0.732177	-0.038863
C	-1.613651	0.238485	1.256739
C	-1.968192	0.204753	-1.193303
C	-2.877020	-0.831222	-1.034107
C	-2.539325	-0.793113	1.384563
H	-1.711037	0.595827	-2.171786
H	-1.153753	0.667006	2.139154
C	-3.158348	-1.334668	0.248629
H	-3.355710	-1.265672	-1.905652
H	-2.773445	-1.180541	2.370630
H	-3.865702	-2.151307	0.361489
Cl	1.622264	-1.188958	-0.085069
O	2.851900	-1.914690	-0.414895
O	1.530280	-0.969184	1.371302
O	1.641320	0.149009	-0.749523
O	0.441084	-1.920956	-0.556055

Nitrosocarbinolic cation (**1**) - perchlorate contact ion pair (**CIP-p2**):

C	-0.143911	2.600328	-0.707997
H	-0.387221	1.965736	-1.555506
H	0.741576	3.219003	-0.883586
O	-1.260270	3.287916	-0.329047
N	0.282274	1.639049	0.414794
H	-1.014731	3.983844	0.292448
O	-0.217941	1.825855	1.513222
C	1.225608	0.630438	0.196365
C	1.786435	0.472987	-1.089053
C	1.568826	-0.204795	1.281228
C	2.505994	-1.205624	1.066150
C	2.723277	-0.538519	-1.272430
H	1.104891	-0.051816	2.248349
H	1.520092	1.116910	-1.918463
C	3.077159	-1.379036	-0.206200
H	2.789337	-1.860589	1.883623
H	3.173395	-0.677964	-2.249703
H	3.806827	-2.168073	-0.364700
Cl	-1.602053	-1.236601	-0.111729
O	-1.666445	0.130337	-0.683102
O	-0.475647	-1.959751	-0.724886
O	-1.383273	-1.119245	1.342361
O	-2.863365	-1.944219	-0.376674

Transition structure for proton transfer to perchlorate within **1**,perchlorate CIP (**TS-p**):

C	1.573894	0.505082	0.207672
C	1.556094	-0.602011	1.063676
C	2.478947	0.640709	-0.851194
N	0.588788	1.522994	0.437932
C	3.404095	-0.388468	-1.051032
C	2.484515	-1.618947	0.835804
H	0.818442	-0.660026	1.857146
H	2.502131	1.535145	-1.465169
O	0.248453	1.792883	1.607036
C	-0.176467	1.982700	-0.629875
C	3.404254	-1.515191	-0.218070
H	2.484538	-2.495865	1.475898
H	4.131705	-0.300989	-1.851891
H	0.399498	2.156198	-1.540602
O	-1.057286	2.974733	-0.334737
H	4.124248	-2.310455	-0.387052
H	-1.471926	2.758440	0.515111
H	-0.739511	0.863287	-0.936939
Cl	-2.029441	-0.929184	-0.115063
O	-1.307379	-0.281802	-1.317173
O	-1.464929	-2.255614	0.074335

O	-1.747908	-0.052263	1.027409
O	-3.448719	-0.982163	-0.421601

Nitrosocarbinolic cation (**1**) - perchlorate solvent shared ion pair (**SSIP-p**):

C	1.496525	0.424179	0.144511
C	2.255311	-0.305888	1.084029
C	1.374727	0.030507	-1.204691
N	0.834727	1.575916	0.585654
C	2.037265	-1.126591	-1.606100
C	2.906194	-1.452422	0.651268
H	2.330581	0.041083	2.108793
H	0.775586	0.583496	-1.917924
O	0.793416	1.877867	1.768107
C	0.188165	2.558946	-0.397909
C	2.792032	-1.869185	-0.687049
H	3.501636	-2.029100	1.351641
H	1.954556	-1.456285	-2.636678
H	1.043224	3.042893	-0.881854
O	-0.646217	3.406559	0.276760
H	3.301791	-2.771017	-1.014113
H	-0.124964	4.071762	0.741887
H	-0.395194	1.961539	-1.096422
O	-1.335224	0.845928	-2.701297
H	-1.663514	0.078710	-2.205354
H	-2.109481	1.188919	-3.156902
Cl	-1.700626	-1.113956	0.456260
O	-1.872983	-1.322615	-1.004760
O	-1.503918	0.330282	0.706563
O	-2.901259	-1.583441	1.157403
O	-0.514811	-1.850007	0.915545

Transition structure for proton transfer to water within **1**,perchlorate SSIP (**TS-wp**):

C	-1.544233	0.836443	-0.175689
C	-1.685245	1.636496	0.964673
C	-0.683454	1.152520	-1.231963
N	-2.345579	-0.354183	-0.248955
C	0.072055	2.323882	-1.127388
C	-0.908160	2.791958	1.049960
H	-2.363960	1.339801	1.758475
H	-0.640561	0.539522	-2.125249
O	-3.524443	-0.320591	0.144054
C	-1.720172	-1.546622	-0.574967
C	-0.034549	3.136922	0.007211
H	-0.981960	3.422726	1.930596
H	0.735092	2.602554	-1.939957
H	-0.885749	-1.401958	-1.269269
O	-2.620223	-2.551113	-0.817236
H	0.561282	4.042216	0.079892
H	-2.116349	-3.364457	-0.944021
H	-1.093729	-1.712002	0.532883
O	-0.364538	-1.951726	1.655553
H	-0.852280	-1.765170	2.470133
H	0.386352	-1.305252	1.618304
Cl	2.355220	-0.477530	-0.045979
O	2.910913	0.780919	-0.544490
O	1.543801	-0.188101	1.178772
O	3.429090	-1.415104	0.299767
O	1.457242	-1.078069	-1.048730

Nitrosocarbinolic cation (**1**) - water adduct (**w-ncc**):

C	-0.421322	0.060669	-0.331066
C	-1.223212	-1.062466	-0.624731
C	-0.951208	1.254102	0.202972
N	0.955117	-0.030472	-0.571422
C	-2.324266	1.316113	0.425324
C	-2.587997	-0.972524	-0.385488
H	-0.769339	-1.966530	-1.015449
H	-0.329395	2.115633	0.415768
O	1.441787	-0.975236	-1.167596
C	1.929080	1.062249	-0.093613
C	-3.138527	0.209919	0.140345
H	-3.227197	-1.824164	-0.593495
H	-2.759534	2.225889	0.824879
H	1.734523	1.909238	-0.758340
O	3.206651	0.586022	-0.129742
H	-4.206967	0.268391	0.326002
H	3.535988	0.609672	-1.036274
H	1.648066	1.263077	0.936679
O	1.261256	-1.214806	1.814749
H	2.093185	-1.468817	2.228029
H	0.622009	-1.295299	2.530404

Transition structure for proton transfer to water within **1**,water adduct (**w-TS**):

C	-0.512458	0.285618	-0.230726
C	-1.206895	-0.644810	-1.011049
C	-1.111760	1.013843	0.802439
N	0.878275	0.505902	-0.545104
C	-2.466675	0.782130	1.063723
C	-2.554961	-0.864800	-0.723322
H	-0.693265	-1.184123	-1.800780
H	-0.563717	1.775543	1.347531
O	1.232294	0.536435	-1.739108
C	1.797069	0.496626	0.480540
C	-3.183994	-0.155351	0.310802
H	-3.113783	-1.592316	-1.303834
H	-2.960752	1.347375	1.847803
H	1.392529	0.868706	1.425495
O	3.038076	0.901383	0.080586
H	-4.234518	-0.329106	0.523883
H	3.593877	0.966372	0.867581
H	1.811462	-0.818389	0.701917
O	2.042835	-2.061055	0.974320
H	2.292836	-2.567637	0.185661
H	1.259809	-2.501383	1.339637

Hydroxamic acid (**3**) (**3-HA**):

C	-2.018776	-0.576205	-0.449310
H	-1.582683	-1.361282	-1.051418
O	-3.343828	-0.425451	-0.332719
N	-1.241645	0.273521	0.176419
H	-3.768954	-1.125111	-0.844105
O	-1.671446	1.279279	0.867192
C	0.189291	0.084817	0.066428
C	0.756866	-1.152160	0.380678
C	0.964247	1.184144	-0.311315
C	2.349050	1.023567	-0.410450
C	2.146117	-1.298786	0.273987
H	0.482454	2.132247	-0.527411
H	0.130051	-1.973857	0.714889
C	2.940493	-0.214840	-0.118777
H	2.966582	1.864096	-0.713690
H	2.604763	-2.252742	0.517369
H	4.017770	-0.331841	-0.193797

Hydroxamic acid (**4**) (**4-HA**):

C	-2.125752	-0.748137	-0.402554
H	-1.661990	-1.625702	-0.865953
O	-3.338787	-0.516720	-0.372808
N	-1.232635	0.100585	0.152348
O	-1.750245	1.287780	0.666642
C	0.176918	0.034254	0.074512
C	0.814739	-1.185077	0.341748
C	0.911303	1.183092	-0.250798
C	2.303950	1.093370	-0.336538
C	2.205928	-1.266270	0.215345
H	0.395826	2.119590	-0.435564
H	0.233255	-2.049701	0.647661
C	2.953794	-0.128219	-0.111797
H	2.880926	1.979629	-0.585015
H	2.705180	-2.210713	0.411871
H	4.035384	-0.191353	-0.187951
H	-2.693165	1.207605	0.418335

water:

O	0.000000	0.000000	0.119640
H	0.000000	0.752496	-0.478561
H	0.000000	-0.752496	-0.478561

H3O+:

O	0.000000	0.000157	0.000000
H	0.951211	0.178763	0.000000
H	-0.631512	0.733399	0.000000
H	-0.319699	-0.913419	0.000000

ClO4-:

Cl	0.000000	0.000000	0.000000
O	0.851225	0.851225	0.851225
O	-0.851225	-0.851225	0.851225
O	-0.851225	0.851225	-0.851225
O	0.851225	-0.851225	-0.851225
